# Complex assembly from planar and twisted π-conjugated molecules towards alloy helices and core-shell structures

**DOI:** 10.1038/s41467-018-06489-3

**Published:** 2018-10-19

**Authors:** Yilong Lei, Yanqiu Sun, Yi Zhang, Hongyang Zhang, Haihua Zhang, Zhengong Meng, Wai-Yeung Wong, Jiannian Yao, Hongbing Fu

**Affiliations:** 1 Department of Chemistry, School of Sciences, Tianjin University, Tianjin, 300072 P.R. China; 2 Institute of Molecular Functional Materials and Department of Applied Biology and Chemical Technology, The Hong Kong Polytechnic University, Hung Hom, Hong Kong P.R. China; 3 Department of Chemistry, Hong Kong Baptist University, Waterloo Road, Kowloon Tong, Hong Kong P.R. China; 4 Institute of Chemistry, Chinese Academy of Sciences (ICCAS), Beijing, 100190 P.R. China

## Abstract

Integrating together two dissimilar π-conjugated molecules into controlled complex topological configurations remains a largely unsolved problem owing to the diversity of organic species and their respective different assembly features. Here, we find that two structurally similar organic semiconductors, 9,10-bis(phenylethynyl)anthracene (BA) and 5,12-bis(phenylethynyl)naphthacene (BN), co-assemble into two-component helices by control of the growth kinetics as well as the molar ratio of BA/BN. The helical superstructures made of planar and twisted bis(phenylethynyl) derivatives can be regarded as (BA)_*x*_(BN)_1−*x*_ alloys, which are formed due to compatible structural relationship between BA and BN. Moreover, epitaxial growth of (BA)_*x*_(BN)_1−*x*_ alloy layer on the surface of BA tube to form BA@(BA)_*x*_(BN)_1−*x*_ core-shell structure is also achieved via a solute exchange process. The precise control over composition and morphology towards organic alloy helices and core-shell microstructures opens a door for understanding the complex co-assembly features of two or more different material partners with similar structures.

## Abstract (web summary)

Manipulating the assembly of π-conjugated organic molecules into alloys to control composition and shape remains a largely unsolved problem. Here the authors show the co-assembly of two structurally similar organic semiconductors into two-component helices by control of their growth kinetics as well as the molar ratio of the building blocks.

## Introduction

One of the key issues in nanoscience and nanotechnology is to precisely control the dimensions, shapes and compositions of the nanostructures due to the growing technological requirements. To date, many efforts have been directed towards the morphology control of low-dimensional inorganic nanomaterials with tunable compositions and desired spatial distribution, such as noble metal and II-VI semiconductor nanocrystals^1–8^. As a consequence, the resulting superior optical and electrical properties of these nanomaterials render them to be promising candidates for use in optoelectronic and thermoelectric devices^9–13^. Especially, inorganic alloy and core-shell nanomaterials have attained great attention and the solution-processed high-temperature synthesis is demonstrated to be a simple yet efficient method to achieve these nanomaterials^14–18^.

Likewise, it can be expected that organic alloy and core-shell structures are also of particular interest in view of the diversity of organic species and their wide-ranging functional properties. By definition, organic alloys are described as homogeneous solid mixtures formed by random occupancy of an isostructural guest at the crystal lattice of a host over a wide composition range (0 < *x* < 1)^19–25^. However, organic alloys are considerably different from doped organic nanocrystals or organic cocrystals, as depicted in the previous reports^26–36.^ Especially, doped organic nanocrystals are formed by dispersion of a dopant into a host in a low doping ratio^26–30^, while organic cocrystals are constructed by integrating together the constituent materials in a definite stoichiometric ratio via supramolecular interactions, such as hydrogen-bonding^31^ and charge-transfer (CT) interactions^32–36^. For organic alloys, such an enormous and continuous change in material composition could offer improved and/or unexpected optoelectronic properties. A representative case is to integrate together two structurally similar porphyrin donors into a molecular alloy, leading to a dramatically enhanced power conversion efficiency (PCE) for the organic solar cells (OSCs)^37^. Besides the promising applications in OSCs, organic alloys are specially designed to achieve ambipolar electronic performances as well as tunable emission colors^38,39^. Moreover, organic core-shell structures are also of importance owing to the enhanced charge carrier density at the core/shell interfaces^40^. Hence, great effort has been made to manipulate the assembly of π-conjugated organic molecules into alloy and core-shell structures considering their potential applications in optoelectronic devices. However, the composition and shape control towards this aim remains a largely unsolved problem because of the great difficulty in selecting appropriate material pairs with compatible structures and regulating their growth kinetics in a rational manner.

To overcome the synthetic problems, some requirements should be first considered: (1) Two constituent materials have highly similar structures, sizes, and physicochemical properties, which will enable efficient incorporation of a guest into a host crystal over a wide composition range; (2) Epitaxial growth of one crystal on the existing seed could be achieved by tuning the interfacial energy between them. Here, we develop a simple and convenient solution-phase strategy to realize co-assembly of planar and twisted bis(phenylethynyl) derivatives, i.e., BA and BN. Two-component helices comprising BA and BN are achieved during the co-assembly process depending on their molar ratio and crystallization rates, regardless of the adopted solvents. The well-matched molecular structures of BA and BN enable the occupancy of twisted BN molecules at the sites of BA, leading to the formation of (BA)_*x*_(BN)_1-*x*_ alloys. In view of perfect lattice matching between BA and its alloys with different BA/BN molar ratio, we also present a facile solute exchange approach for epitaxial growth to afford BA@(BA)_*x*_(BN)_1-*x*_ core-shell microstructures, in which BA tubes act as seeds and are accompanied by the formation of (BA)_*x*_(BN)_1-*x*_ shell layer. The present findings can serve as a perfect example for understanding the formation mechanism of complex topological configurations made of π-conjugated organic molecules.

## Results

### Co-assembly of BA and BN

**Fig. 1:**
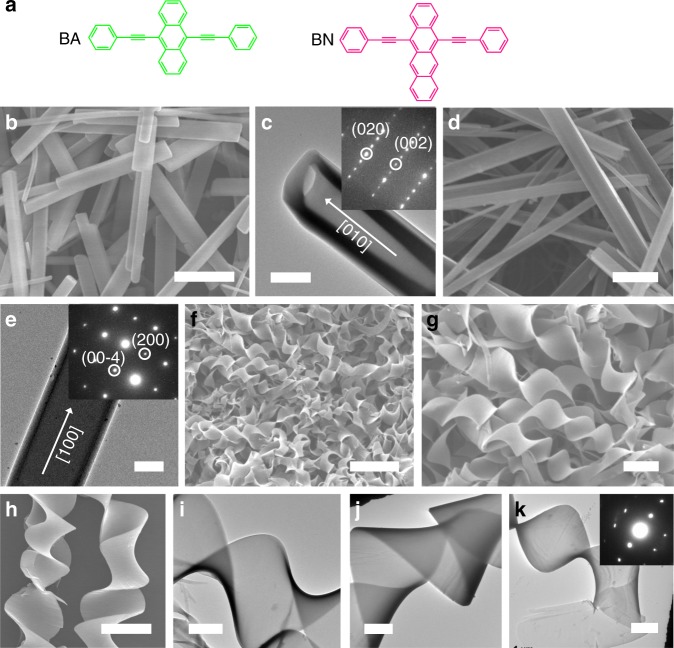
Molecular design and morphology characterization of BA, BN, and BA/BN helical assemblies. **a** Schematic showing the molecular structures of BA (green) and BN (pink). **b**, **d**, **f**, and **g** SEM images of (**b**) BA tubes, (**d**) BN tubes, and BA/BN helical ribbons obtained at m/m = 2:1 and v/v = 4:1 at (**f**) low and (**g**) high magnification. Scale bars, 10 μm in **b**, **d**, **g** and 30 μm in **f**. TEM images of single **c** BA and **e** BN tubes. Scale bars, 2 μm in **c** and 200 nm in **e**. Insets show the selected-area electron diffraction (SAED) patterns of BA and BN tubes, respectively. **h** SEM and **i**, **j** TEM images of single (**i**) left- and (**j**) right-handed helical ribbons. Scale bars, 10 μm in **h** and 30 μm in **i**, **j**. **k** TEM image of single typical helix. Inset shows the corresponding SAED pattern. Scale bar, 2 μm

We selected two π-conjugated semiconductors of BA and BN (Fig. 1a) to study the possibility of constructing (BA)_*x*_(BN)_1-*x*_ alloys considering their minor differences in structures and sizes and obviously different luminescent features. For comparison, both BA and BN assemblies were first obtained by a liquid-phase assembly method^31,32^. Specifically, 1 mL of pure BA (C_BA_ = 5 mM) or BN (C_BN_ = 2.5 mM) solution in THF was rapidly injected into 5 mL of ethanol/H_2_O (v/v) mixtures to induce self-assembly of BA or BN. By means of the SEM (Fig. 1b) and TEM (Fig. 1c) tests, we can see that BA crystallizes into 1D rectangular microtubes at v/v = 9:1. Similarly, tubular BN assemblies (Fig. 1d, e) were also achieved at 4:1 (v/v). The sizes of 1D BA and BN assemblies (Supplementary Figs. 1, 2) can be further tailored by continuously adjusting v/v. Similar to the cases of pure BA or BN, we also attempted to explore the co-assembly of BA and BN by adding a stock solution (1 mL) of BA/BN in THF with a 2:1 molar ratio (C_BA_ = 5 mM, C_BN_ = 2.5 mM, m/m) into 5 mL of an 4:1 ethanol/H_2_O mixture. Instead of straight 1D structures, giant helical microribbons with homogeneous appearances (Fig. 1f–h) were achieved within a few minutes. During this process, left- and right-handed helices (Fig. 1h) were formed simultaneously, which were further confirmed by in-vivo tracking the formation processes of helical ribbons using laser confocal scanning microscopy (Supplementary Movies 1–3). Specifically, the helices with opposite handedness would emerge at the initial growth stage and then grow rapidly towards the initial direction, which are followed by continuous twisting. One can also observe that each individual helix has an average thickness of 400 nm, a width of 10 μm, and a length of tens of micrometers. TEM images again reveal the formation of helical configurations including either left- (Fig. 1i) or right- (Fig. 1j) handed helices, suggesting that the final product should be a racemic mixture because achiral BA and BN were used as the building blocks^41–44^. The helical ribbons deposited on a quartz substrate were relatively stable and kept their original morphology even upon exposure to daylight for two weeks (Supplementary Fig. 3).

Naturally, we also expect to modulate the co-assembly morphologies of BA and BN by continuously varying v/v while m/m = 2:1 was kept unchanged. At v/v = 17:3, 1D ribbons are stacked together to form flower-like helical superstructures (Supplementary Fig. 4a) after ten minutes. Twisted ribbons as well as a small amount of helical ribbons (Supplementary Figs. 4b, 5) were obtained upon decreasing v/v to 3:1. By further decreasing v/v to 7:3, the middle parts of the resultant ribbons would curl into helices, while the end parts are almost straight (Supplementary Fig. 4c). Besides the helical ribbons formed at m/m = 2:1, BA/BN co-assemblies were also synthesized by precisely tuning m/m from 100:1 to 4:1. Consequently, thick microtubes would transform into straight nanoribbons (Supplementary Fig. 6). The dependence of BA/BN co-assemblies on the solvent/molar ratio is summarized in Supplementary Table 1. Instead of THF solvent, both DMF and acetone were also used as good solvents to explore the co-assembly of BA and BN. Similar to the cases using THF as a good solvent (Fig. 1f–h), BA/BN helices (Supplementary Figs. 7, 8) were also achieved readily. Thus, the formation of the helical assemblies is dependent on the molar ratio and crystallization rates of BA/BN mixtures, and independent of the good solvents.

### Structural analysis of (BA)_0.72_(BN)_0.28_ alloy helices

Notably, it is unlikely to achieve such a huge morphology transition from microtubes to helical microribbons by doping a small ratio of BN into BA host^26–30^. Hence, the co-assembled helices may be regarded as (BA)_*x*_(BN)_1-*x*_ alloy or BA-BN co-crystal. As described previously^38,45^, the exact molar ratio of the two components in the organic alloys was determined by dissolving each alloy and comparing the integrals of the corresponding ^1^H NMR peaks from the two materials. Interestingly, the present ^1^H NMR data (Supplementary Figs. 9–11) reveal that the above helical ribbons obtained at m/m = 2:1 and v/v = 4:1 correspond to (BA)_0.72_(BN)_0.28_. In contrast to the helical structures, the co-assembled morphologies formed at m/m = 4:1 and 20:1 correspond to (BA)_0.8_(BN)_0.2_ and (BA)_0.94_(BN)_0.06_ (Supplementary Figs. 12, 13), respectively. We hereby conclude that the BA/BN co-assemblies with variable compositions should be derived from (BA)_*x*_(BN)_1-*x*_ alloy rather than BA-BN co-crystal.

**Fig. 2:**
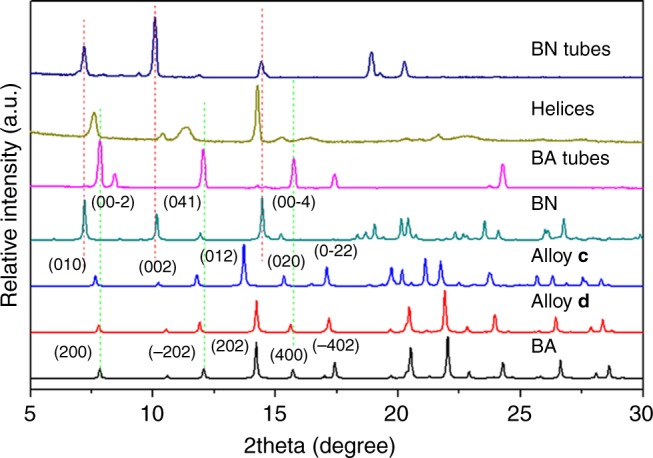
XRD characterization of BA, BN, and BA/BN helical assemblies and the corresponding single crystals. Powder XRD patterns of BA tubes, BN tubes, and (BA)_0.72_(BN)_0.28_ helices and simulated patterns from the single crystals of BA, BN, alloys **c** and **d**. The green and orange dashed lines represent the peaks of BA and BN, respectively

To obtain the detailed structural information of the helical assemblies, we first performed the powder XRD analysis of (BA)_0.72_(BN)_0.28_ helices and their constituent materials (Fig. 2). Typically, the XRD patterns of BA tubes match well with the simulated result of α-form BA crystal^46–48^. BN single crystal was also grown by slow evaporation of a stock solution of BN in THF, which can be used to recognize the XRD patterns of BN tubes. The SAED patterns of BA and BN tubes (insets in Fig. 1c, e) can be clearly indicated by means of the simulated XRD patterns from their single crystals, indicating that both of them have single-crystalline structures and they were grown along [010] and [100] directions, respectively. In contrast to the pure BA or BN, the XRD patterns of (BA)_0.72_(BN)_0.28_ helices exhibit the formation of a new crystal phase. Moreover, it is apparent that the helices are still highly crystalline although new broad peaks such as 7.6^°^ and 10.4^°^ would appear, also revealing the alloy nature of the crystal phase. The SAED pattern of single typical helix further confirmed the single-crystalline nature of (BA)_0.72_(BN)_0.28_ helices (Fig. 1k). At m/m = 2:1, the helices obtained at v/v = 3:1 and 7:3 have almost the same crystal structures as those formed at v/v = 4:1, as verified by the corresponding XRD patterns (Supplementary Fig. 14). Hence, the BN content in the co-assemblies shows a minor change when their morphologies change under the present conditions.

Besides slight peak shifts, the XRD patterns of BA/BN co-assemblies formed at m/m = 100:1 and 20:1 (Supplementary Fig. 15) are almost consistent with those of the pure BA, indicating that the incorporation of a small amount of BN guest has a minor effect on the crystal structure of the BA host. At m/m = 4:1, new XRD peaks from the straight nanoribbons (Supplementary Fig. 15) would emerge and are close to the cases of (BA)_0.72_(BN)_0.28_ helices obtained at m/m = 2:1 (Fig. 2). Thus, it seems reasonable that the XRD patterns of BA/BN co-assemblies gradually shift towards lower positions of BN component upon decreasing m/m. The present results clearly demonstrated that (BA)_x_(BN)_1-x_ alloys rather than BA-BN cocrystals^31–36^ or mixed phases of BA/BN^45^ were formed upon co-assembly of BA and BN. That is, BN molecules would locate themselves randomly at the sites provided by the BA host^49^.

To further clarify the molecular packing of (BA)_*x*_(BN)_1-*x*_ alloys, a series of alloy single crystals with variable compositions (alloys **a**–**d**) were synthesized by slow evaporation of two different preparative solutions of BA/BN with m/m = 2:1. Typically, alloys **a** and **b** were grown from THF, whereas alloys **c** and **d** were obtained from DMF. The crystallographic data for the resultant large-sized alloys (Supplementary Table 2) are closely related to those of α-form BA^46–48^. Similar to the cases of ternary diarylethene-based alloys grown from ethanol and hexane^50^, the unit cell parameters of alloys **a**–**d** also show small changes due to the differences in crystallization rates and solubilities of BA/BN in THF and DMF. Compared with alloys **a**, **b** and **d**, alloy **c** displays larger structural change because it contains a more BN content. Moreover, we find that the molar ratio of BN incorporated in alloy **c** would be significantly less than that in the co-assembled helices, as verified by the XRD results of (BA)_0.72_(BN)_0.28_ helices and alloy **c** (Fig. 2). Although great effort has been made, it is still difficult to achieve a well-matched alloy single crystal with (BA)_0.72_(BN)_0.28_ helices. However, alloys **c** and **d** can yet be regarded as desired model crystals to simulate the molecular packing of (BA)_*x*_(BN)_1-*x*_ helices.

**Fig. 3:**
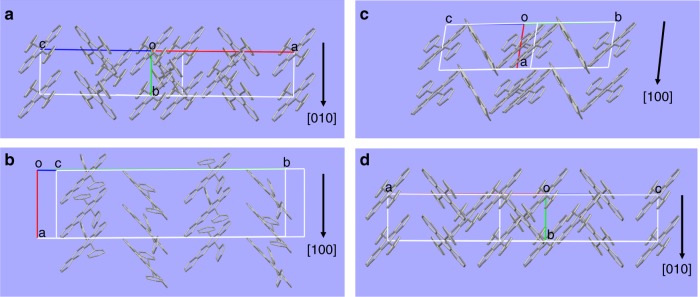
Structural analysis of BA, BN, alloys **c** and **d**. **a**–**d** Molecular packing of single crystals of **a** BA, **b** BN, **c** alloy **c** and **d** alloy **d** along π-π stacking directions

For comparison, the molecular packing of pure BA or BN was first revealed. For BA crystal (Fig. 3a), its constituent molecules show nearly planar structures with a herringbone angle of around 80.5^°^ and a π-π stacking distance between two closest BA molecules is about 3.349 Å. Besides the π-π interactions along the b axis, there are also C-H···π interactions between BA and its four neighbor molecules. As a consequence, a long-range π-π stacking interaction leads to the 1D assembly morphology. That is, rod-like BA crystals were grown along the [010] direction, which is consistent with the theoretical calculated result (Supplementary Fig. 16) simulated by Material Studio based on the optimized energy consideration^46–48^. For BN crystal (Fig. 3b), the torsion angles between naphthacene and two phenylethynyl groups are calculated to be 29.5^°^ and 32.5^°^, respectively. Moreover, the π-π stacking distance between two closest naphthacene groups along [100] direction is estimated to be about 3.23 Å. Similar to that of BA, the π-π stacking interaction in BN crystal should also be responsible for the formation of 1D morphology with [100] growth direction (Supplementary Fig. 17).

On the basis of the definition of organic alloys, it will be hard to recognize BN molecules from the crystal lattice of BA in alloys **c** and **d** because the random occupancy of BN at the sites of BA host represents a disordered structure at the molecular scale^38,45^. Nevertheless, they can still be regarded as long-range ordered single crystals^49,50^. As expected, the crystal structures of alloys **c** (Fig. 3c) and **d** (Fig. 3d) both contain only BA molecules and exhibit similar molecular configurations relative to pure BA. Hence, it seems reasonable that planar BA and twisted BN molecules in these alloys are stacked together via strong π-π stacking. The π-π stacking distances between two closest BA molecules or BA and BN molecules in alloys **c** and **d** are estimated to be 3.466 and 3.367 Å, respectively. Thus, these values are slightly greater than that of pure BA along [010] direction (3.349 Å) upon incorporation of BN into BA host. Also, we infer that the π-π stacking interactions in alloys **c** and **d** would favor the formation of 1D morphologies, as confirmed by the related structural simulations (Supplementary Figs. 18, 19). The growth directions of the 1D assemblies derived from alloys **c** and **d** were determined to be [100] and [010], respectively.

### Formation mechanism of (BA)_0.72_(BN)_0.28_ alloy helices

**Fig. 4:**
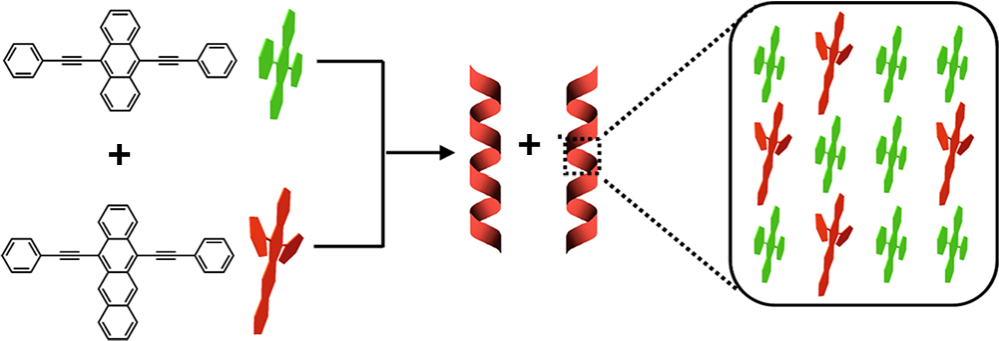
Schematic illustration of the molecular packing of (BA)_0.72_(BN)_0.28_ alloy helices. In (BA)_0.72_(BN)_0.28_ alloy helices, the BA host molecules are randomly replaced by BN molecules and a BA-BN dimeric structure with a slight twist is formed

Undoubtedly, well-matched structural relationship between BA and BN is essential to the formation of (BA)_0.72_(BN)_0.28_ alloy helices, as shown in Fig. 4. Upon stacking BA and BN together, the BA host would provide the sites in which BN can occupy, thus generating a less tightly π-π stacking interaction between a BA-BN dimeric structure than that between two BA molecules (BA-BA) due to their remarkable difference in the intramolecular torsion angles. The enlarged π-π stacking distances in alloys **c** and **d** relative to pure BA or BN confirm this conclusion. As already described^42,43^, such a BA-BN dimeric structure with a slight twist rather than parallel stacking may be suitable to maximize their interactions. To get more information about the molecular orientation of BN in the BA crystal, an ONIOM (B3LYP/6-31 G (d,p):UFF) approach was used to optimize the geometries built by doping BN into BA crystal (Supplementary Fig. 20), as reported previously^51^. The simulated results suggest that the most stable structure of BN in the BA host has much smaller dihedral angles between the naphthacene and two phenylethynyl groups, compared to those in the pure BN crystal. The change in torsion angles may be due to the confinement effect of the rigid BA host, also giving a BA-BN dimeric structure with a slight twist in the active region. Similar to the case of BN doped BA, the geometries of BA doped BN were also built to reveal the intermolecular interaction between BA and BN (Supplementary Fig. 21). As expected, the dihedral angles between the anthracene and two phenylethynyl groups for the BA guest would increase owing to the repulsive force between BA and BN. In order to reduce the total free energy in our co-assembly systems, consistently twisting the BA-BN dimeric units towards the same direction would be a preferable strategy (Supplementary Movies 1–3), thereby leading to helical superstructure^42,43^. Moreover, an appropriate composition range containing m/m from 2:1 to 1:1 would also be necessary to realize helical structures (Fig. 1f–j, Supplementary Figs. 22, 23), possibly because large amounts of BA-BN dimeric units are needed to generate larger twisting forces.

Besides the structural compatible BA/BN pair and appropriate molar ratio, the control of growth kinetics is also crucial to achieve the helical structures. Typically, (BA)_*x*_(BN)_1-*x*_ helices were obtained by adding a series of stock solutions (1 mL) of 2:1 BA/BN mixture in THF into 5 mL of ethanol/H_2_O mixtures at v/v = 17:3, 4:1, 3:1, and 7:3. In the absence of BA, pure BN self-assembles to tubular 1D aggregates at v/v ≤ 4:1. Thus, the efficient incorporation of BN into the BA host to form (BA)_*x*_(BN)_1-*x*_ helices at the lower water content (v/v > 4:1) is attributed to the similarity of BA and BN in terms of molecular structures and sizes. In contrast to the helical superstructures, straight nanoribbons (Supplementary Fig. 24) were formed upon diffusion of 1 mL of 2:1 BA/BN solution in THF into 5 mL of ethanol/H_2_O mixtures at v/v = 13:7. Helical ribbons (Supplementary Fig. 25) were still achieved by adding lower concentrations of BA and BN in THF with a 2:1 molar ratio (C_BA_ = 2.5 mM, C_BN_ = 1.25 mM) into an ethanol/H_2_O mixture at the same v/v. The above results further confirm that co-assembled morphologies of (BA)_*x*_(BN)_1-*x*_ alloys were determined depending on the crystallization rate of BA/BN mixtures, i.e. kinetic control. Upon mixing the BA/BN solutions with ethanol/H_2_O mixtures at v/v = 17:3, 4:1, 3:1, and 7:3, it is inferred that BA-BN dimeric units in the solvent mixtures containing low water contents (e.g., v/v = 4:1) would arrange themselves into ordered configurations with minimized overall energy, thus generating helical ribbons. In the solvent mixtures containing more water content (e.g., v/v = 13:7), BA-BN units have no enough time to afford an optimized twisted configuration due to the stronger hydrophobic effect between BA-BN pair and water. Hence, straight ribbons were still obtained as a consequence of π-π stacking interactions in the alloys. We also tried to probe what happens when BN is replaced by a symmetrically substituted naphthacene derivative, 5,6,11,12-tetraphenylnaphthacene (rubrene). As shown in Supplementary Fig. 26, phase separation would occur upon co-assembly of BA and rubrene, possibly due to the large difference in their molecular structures and sizes.

### Optical properties of (BA)_0.72_(BN)_0.28_ alloy helices

**Fig. 5:**
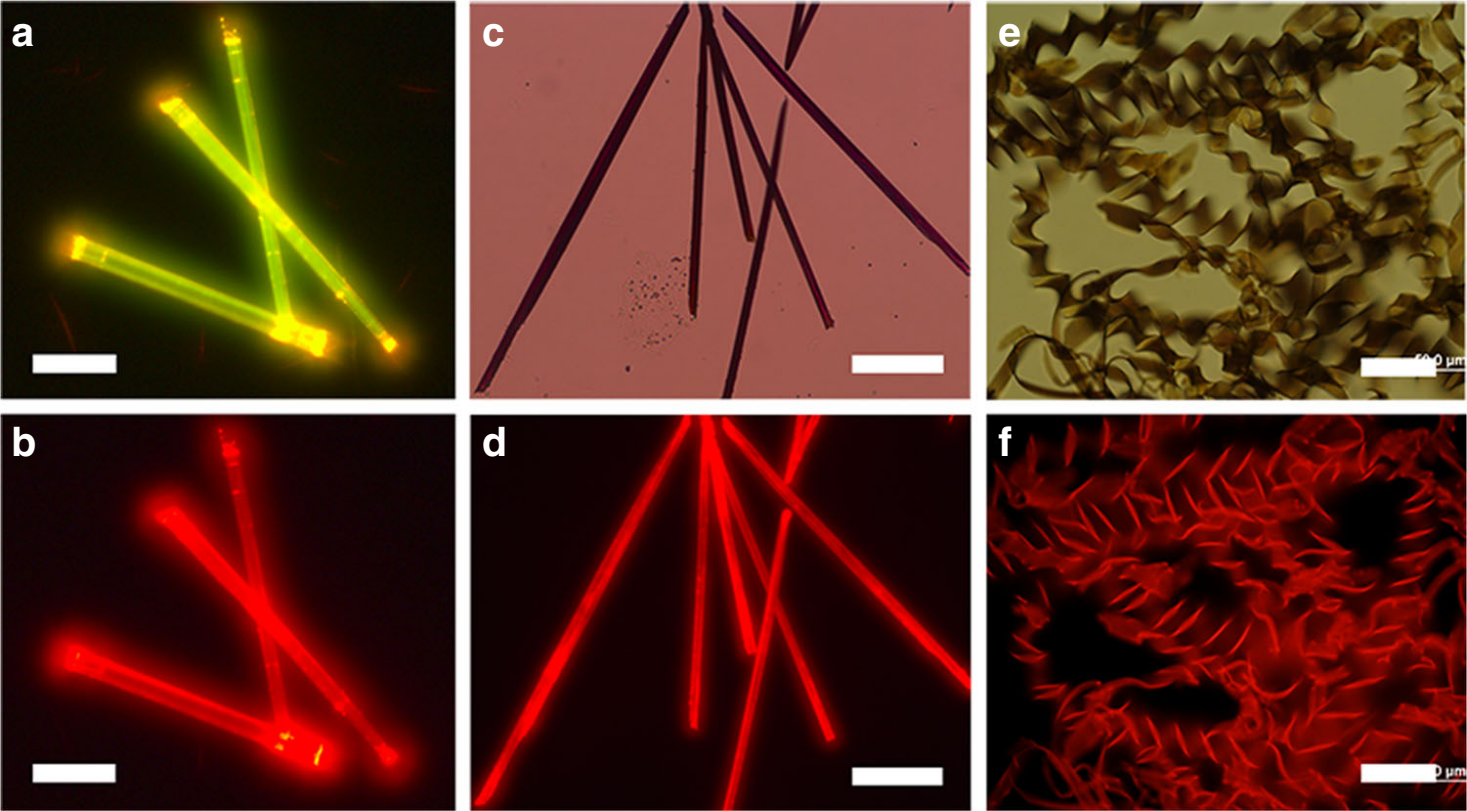
Optical characterization of BA, BN, and (BA)_0.72_(BN)_0.28_ helical assemblies. When excited by (**a**) blue and (**b**, **d** and **f**) green light, fluorescence microscopy images of (**a**, **b**) BA tubes, (**d**) BN tubes, and (**f**) (BA)_0.72_(BN)_0.28_ helices. Scale bars, 25 μm in **a**, **b**, 50 μm in **d**, and 20 μm in **f**. Bright-field optical microscopy images of (**c**) BN tubes and (**e**) (BA)_0.72_(BN)_0.28_ helices. Scale bars, 50 μm in **c** and 20 μm in **e**

Next, the optical and fluorescence microscopy tests were performed to investigate the luminescence features of BA, BN, and (BA)_0.72_(BN)_0.28_ helices (Fig. 5), which can also be used to determine the structural homogeneity of BA/BN helices. As shown in Fig. 5a, b, BA tubes emit strong yellow-green light upon excitation with blue light, which turn into weak red light when excited by green light. BN tubes exhibit dark red light (Fig. 5d) when excited by green light. Similar to that of pure BN crystals, (BA)_0.72_(BN)_0.28_ helical ribbons also emit uniform and bright red light (Fig. 5f) upon excitation with blue or green light. Moreover, (BA)_*x*_(BN)_1-*x*_ alloy assemblies obtained from m/m = 100:1 to 4:1 show tunable emission colors from orange-red to NIR (Supplementary Figs. 27–29), indicating possible energy transfer (ET) processes from BA to BN.

**Fig. 6:**
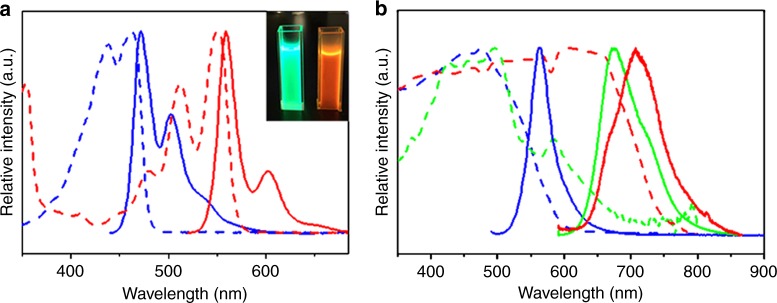
Spectral characterization of BA, BN, and (BA)_0.72_(BN)_0.28_ helical assemblies. **a** Normalized absorption and PL spectra of (blue curves) BA and (red curves) BN monomer solutions in THF. Inset shows the corresponding photographs when excited by 365 nm. **b** Normalized absorption and PL spectra of (blue curves) BA tubes, (green curves) (BA)_0.72_(BN)_0.28_ helices, and (red curves) BN tubes

Spectral measurements of (BA)_0.72_(BN)_0.28_ helical assemblies and its constituent materials were carried out to further study their detailed optical properties. Fig. 6a displays the absorption and photoluminescence (PL) spectra of pure BA and BN monomer solutions in THF. Specifically, multiple resolved absorption bands can be detected in the BA and BN solutions. Upon excitation with 365 nm, their emission colors correspond to the characteristic green (left inset in Fig. 6a) and orange light (right inset in Fig. 6a), respectively. The absorption bands would become less resolved for BA or BN microtubes and a new broad band appears at the longer wavelength due to the aggregation of BA or BN molecules in their assemblies. Similarly, the PL spectra of tubular BA and BN structures (Fig. 6b) also show obvious red-shifts relative to those of their monomer solutions (Fig. 6a). Especially, BA tubes display a single PL band at 560 nm (blue solid curve), which corresponds to yellow or yellow-green light. BN tubes exhibit a main PL band at 710 nm as well as a weak shoulder peak at 660 nm (red solid curve), which corresponds to the NIR light. Similar to that of pure BN tubes, (BA)_0.72_(BN)_0.28_ alloy helices show a main PL peak at 675 nm and an additional weak shoulder peak at 718 nm (green solid curve). Compared with the case of (BA)_0.72_(BN)_0.28_ helices formed at v/v = 4:1, one can observe that the PL spectra of the helices obtained at v/v = 17:3, 3:1, and 7:3 are similar except for the slight red-shifts (Supplementary Fig. 30), suggesting that BN is uniformly incorporated to the BA host in an aggregated state.

In view of the efficient intermixing between BA and BN in (BA)_*x*_(BN)_1-*x*_ alloy assemblies and a good overlap between the PL spectrum of BA and the absorption spectrum of BN (Fig. 6a, b), efficient ET processes from BA to BN should be involved in a series of (BA)_*x*_(BN)_1-*x*_ co-assemblies, giving rise to the emission colors of the alloys towards BN component when decreasing m/m. To confirm these processes, we performed the PL measurements of (BA)_*x*_(BN)_1-*x*_ alloy assemblies obtained at m/m = 100:1, 20:1, and 4:1 (Supplementary Fig. 31). The PL bands derived from BA component in the range of 500-600 nm are almost completely quenched, whereas new PL peaks originated from BN component in the range of 600-800 nm are gradually red-shifted upon decreasing m/m. As a result, their emission colors from orange-red to nearly NIR can be tailored, as further verified by the corresponding fluorescence microscopy results (Supplementary Figs. 27–29). Hence, highly efficient ET processes from self-assembled BA to the monomers or aggregates of BN can occur^52,53^ and the ET efficiency could reach up to 100% below m/m = 100:1. That is, BN guest can be uniformly incorporated into the crystal lattice of the BA host as a possible consequence of their desired compatible crystal structures.

### Synthesis of BA@(BA)_*x*_(BN)_1-*x*_ core-shell structures

To date, however, the successful fabrication of organic heterostructures towards core-shell structures has been also extremely rare considering that perfect lattice matching between the core and shell layers (f < 3%) is hard to satisfy^54^. In fact, phase separation will occur upon coating BN onto BA crystal due to a large lattice mismatch between them (f = 7.87%). That is, it is rather difficult to achieve BA@BN core-shell structures. Yet an available tool to realize this is to integrate BA and (BA)_x_(BN)_1−x_ alloy together, where the interfacial energy between them can be significantly improved, as confirmed by small differences in the XRD patterns of BA and (BA)_0.72_(BN)_0.28_ helices (Fig. 2). For instance, the lattice mismatch between BA and alloy **c** is estimated to be f = 2.28%.

As a proof of concept, we develop a simple solute exchange route for the fabrication of core-shell structures by applying BA tubes as a seed and a saturated supernatant of as-prepared (BA)_0.72_(BN)_0.28_ alloy helices as the shell layer solution. During this process, the self-dissolution of BA tubes dispersed in the saturated solution of (BA)_0.72_(BN)_0.28_ helices can be efficiently improved. As a consequence, the outer layer molecules of BA seed could be partially replaced by (BA)_*x*_(BN)_1−*x*_ alloy derived from the helix supernatant because dynamic assembly and disassembly process for BA crystal always occurs in the above mixtures^55,56^. We thereby infer that epitaxial growth of (BA)_*x*_(BN)_1-*x*_ alloy on the surface of BA tube may also be achieved in the present solute exchange process, similar to a cation exchange strategy towards inorganic core-shell nanostructures^57,58^.

### Structural analysis and optical properties of BA@(BA)_*x*_(BN)_1-*x*_ core-shell structures

**Fig. 7:**
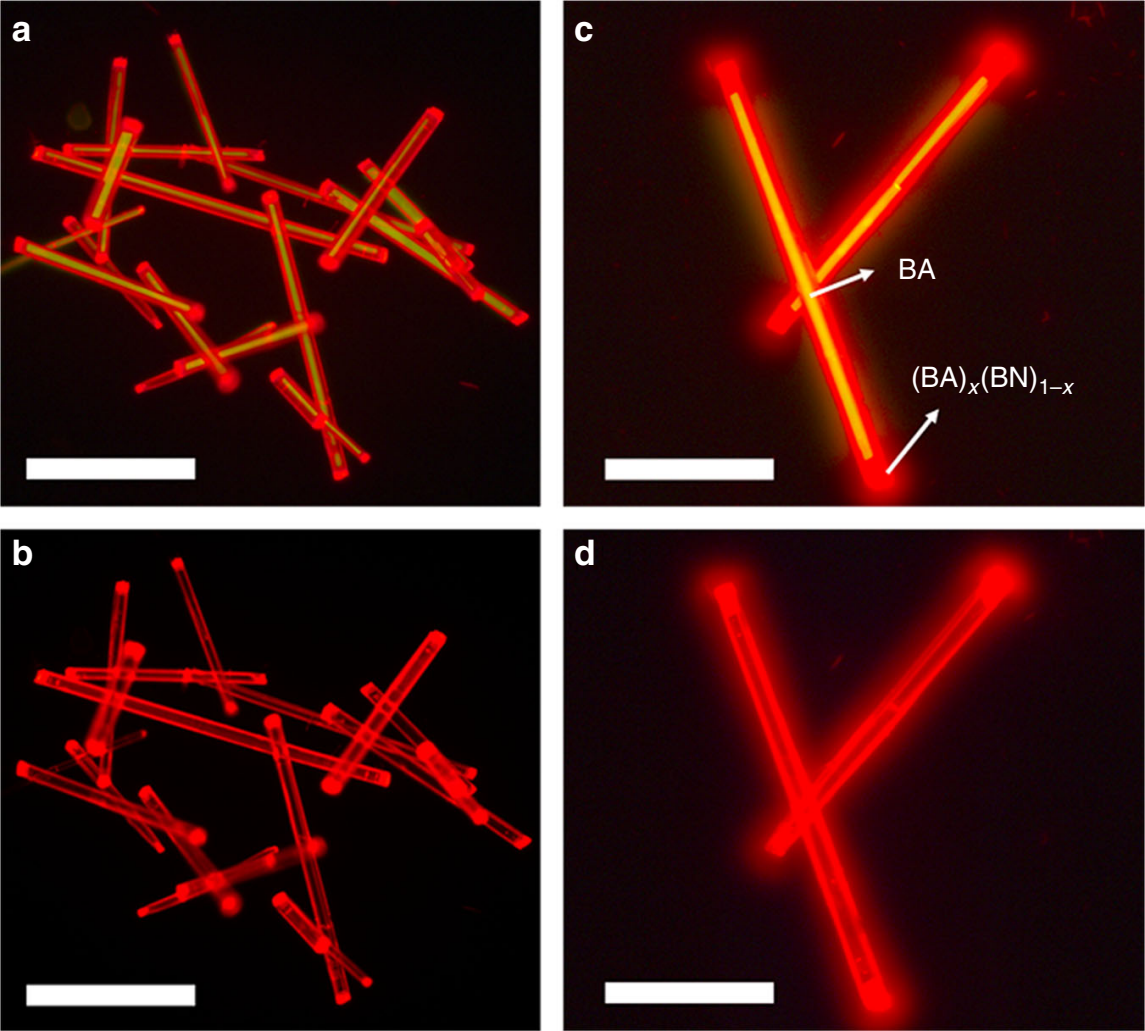
Optical characterization of BA@(BA)_*x*_(BN)_1-*x*_ core-shell structures. **a**–**d** Upon excitation with (**a**, **c**) blue and (**b**, **d**) green light, fluorescence microscopy images of BA@(BA)_*x*_(BN)_1-*x*_ core-shell structures obtained by mixing BA tubes with 2 mL of a saturated supernatant of (BA)_0.72_(BN)_0.28_ alloy helices at (**a**, **b**) low and (**c**, **d**) high magnification. Scale bars, 50 μm in **a**, **b**, and 25 μm in **c**, **d**

As expected, the fluorescence microscopy results shown in Fig. 7a, c demonstrate that a bright red-emitting layer is deposited on a yellow-emitting tube when excited by a blue light, thus suggesting the formation of BA@(BA)_*x*_(BN)_1-*x*_ core-shell structures. Upon excitation with green light, the inner BA tube becomes nearly nonemissive, whereas the (BA)_*x*_(BN)_1-*x*_ shell turns into dark red light (Fig. 7b, d). The corresponding SEM results further verify the uniformity of (BA)_*x*_(BN)_1-*x*_ shell layer (Supplementary Fig. 32). By mixing BA tubes with different amounts of (BA)_0.72_(BN)_0.28_ helix supernatants, such as 1 and 3 mL, the shell thickness of the core-shell microstructures can be varied accordingly (Supplementary Fig. 33). Notably, the relative PL intensity ratio between (BA)_*x*_(BN)_1-*x*_ shell and BA seed would increase gradually upon increasing the thickness of the outer layer (Supplementary Fig. 34), possibly because there is an energy transfer process from BA to (BA)_*x*_(BN)_1-*x*_ across the core-shell interfaces.

**Fig. 8:**
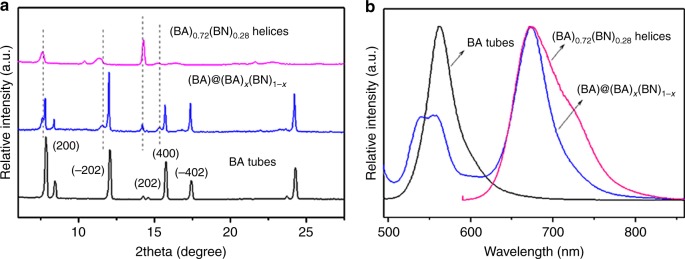
XRD and PL characterization of BA tubes, (BA)_0.72_(BN)_0.28_ helices, and BA@(BA)_*x*_(BN)_1-*x*_ core-shell structures. **a** XRD patterns of BA tubes, (BA)_0.72_(BN)_0.28_, and BA@(BA)_*x*_(BN)_1-*x*_ core-shell structures obtained by mixing BA tubes with 2 mL of a saturated supernatant of (BA)_0.72_(BN)_0.28_ alloy helices. The dashed lines represent the peaks of (BA)_*x*_(BN)_1-*x*_ shell. **b** The corresponding PL spectra when excited by 480 nm

To further investigate the structural information of the core-shell microstructures, we also examined the XRD patterns of BA, (BA)_0.72_(BN)_0.28_, and BA@(BA)_*x*_(BN)_1-*x*_ crystals (Fig. 8a). Obviously, the main peaks of BA@(BA)_*x*_(BN)_1-*x*_ core-shell structures can be indexed to the inner BA tube, whereas some weak peaks, such as 7.6^°^, 11.6^°^, and 15.3^°^ should be derived from (BA)_*x*_(BN)_1-*x*_ shell (dashed lines in Fig. 8a). Nevertheless, small peak red-shifts of (BA)_*x*_(BN)_1-*x*_ shell relative to those of (BA)_0.72_(BN)_0.28_ helices suggest that the shell has a new alloy composition with a lower molar ratio of BN. The corresponding PL spectra were also performed to study the optical properties of the core-shell microstructures, as shown in Fig. 8b. In contrast to that of the pure BA tubes at 562 nm (black line), BA@(BA)_*x*_(BN)_1-*x*_ core-shell structures exhibit three PL bands at 538, 556, and 675 nm (blue line). As described above, (BA)_0.72_(BN)_0.28_ helices show PL peaks above 600 nm (pink line), which indicates that the present PL band at 675 nm is originated from (BA)_*x*_(BN)_1-*x*_ shell, whereas the two splitting peaks at 538 and 556 nm are derived from the BA tubes.

To gain an in-depth understanding of the solute exchange process, we designed a control experiment by mixing pure BA microtubes with 3 mL of saturated supernatants of the as-prepared (BA)_*x*_(BN)_1-*x*_ alloy assemblies obtained at m/m = 20:1 and 4:1, respectively. Clearly, the fluorescence microscopy images shown in Supplementary Figs. 35, 36 display that the shell layers made of (BA)_*x*_(BN)_1-*x*_ alloys with variable compositions were formed on the BA seed when excited by the blue or green light, which are consistent with the cases formed at m/m = 2:1. Moreover, the corresponding PL spectra (Supplementary Fig. 37) also clearly confirm that the luminescence features of the core-shell structures are closely related to the alloy compositions of the outer layers. Hence, the present solute exchange method is demonstrated to be effective to develop BA@(BA)_*x*_(BN)_1-*x*_ core-shell structures. In contrast, (BA)_*x*_(BN_)1-*x*_ cannot be deposited on the surface of BN tube to form BN@(BA)_*x*_(BN)_1-*x*_ core-shell structures upon mixing BN microtubes with saturated supernatant of (BA)_0.72_(BN)_0.28_ alloys, as confirmed by the fluorescence microscopy and SEM results (Supplementary Fig. 38). We infer that the large lattice mismatch between BN and (BA)_0.72_(BN)_0.28_ makes it hard to achieve BN@(BA)_*x*_(BN)_1-*x*_ core-shell structures.

## Discussion

The present work reports kinetic co-assembly of two π-conjugated bis(phenylethynyl) derivatives namely BA and BN where BA acts as a planar configuration and BN as a twisted one. Although different molecular configurations exist in BA and BN, however, both of them can self-assemble into 1D tubular structures in a solvent mixture of THF/ethanol/H_2_O as a consequence of the π-π stacking interactions. In contrast, helical ribbons were formed upon co-assembly of BA and BN in a 2:1 molar ratio, which appear as (BA)_0.72_(BN)_0.28_ alloy as confirmed by the NMR and XRD results. Structural and theoretical analysis further demonstrates that BA serves as a host to provide the sites that can be occupied by the BN component, thus forming a slightly twisted BA-BN dimeric structure. Such co-assembled alloy helices can be realized depending on the well-matched structural design of constituent materials and the precise control of growth conditions. The random occupancy of BN molecules at the sites of BA host enables suitable distance between BA and BN, thus leading to the emission colors ranging from yellow to NIR through the highly efficient ET processes. Similar to the formation of supramolecular block copolymers via co-assembly of planar and core-twisted perylene bisimide derivatives (PBIs **1**/**2**)^59^, the present morphology control towards (BA)_*x*_(BN)_1-*x*_ helices is also determined based on a kinetically controlled pathway. By the same token, it will become possible to construct more types of alloy co-assemblies by rational manipulation of the growth kinetics of π-conjugated constituent materials with specific structural relationships.

Inspired by inorganic core-shell nanostructures developed by a cation exchange method, their organic counterparts were also constructed by a facile solute exchange route. To realize this aim, a prerequisite is that perfect lattice matching between core and shell layers should be fulfilled. Unfortunately, it represents a difficult challenge to access BA@BN core-shell structures due to the larger lattice mismatch between BA and BN. To our surprise, (BA)_*x*_(BN)_1-*x*_ alloy compositions can be continuously varied, which enables the well-matched structural relationship between BA and (BA)_*x*_(BN)_1-*x*_. Moreover, as-prepared BA tubes are dynamically equilibrated upon mixing with the saturated solution of (BA)_0.72_(BN)_0.28_ helices, facilitating the replacement of outer layer molecules of BA tubes by (BA)_*x*_(BN)_1-*x*_. As a result, two-color-emitting BA@(BA)_*x*_(BN)_1-*x*_ core-shell microstructures were formed, which may be used as a general synthetic method to achieve organic core-shell configurations. Importantly, the present results provide a promising platform to explore the effects of the specific structural relationships between two constituent materials on the complex configurations, particularly functional complex structures.

## Methods

### Materials

BA, BN, and rubrene were purchased from Sigma-Aldrich, and used without further treatment. THF (A.R.), ethanol (HPLC grade), DMF (A.R.) and acetone (HPLC grade) were purchased from Beijing Chemical Agent Ltd., China. Ultrapure water with a resistivity of 18.2 MΩ•cm^−1^ was obtained by using a Milli-Q apparatus (Milipore).

### Synthesis of BA and BN microtubes

1D BA microtubes were prepared by a liquid-phase self-assembly route. In a typical synthesis, 1 mL of a stock solution of BA in THF (5 mM) was rapidly injected into 5 mL of (v/v) ethanol/H_2_O mixtures at room temperature and the mixture was kept undisturbed for 10 min, afterwards a yellow suspension was formed. The volume ratio of ethanol and H_2_O (v/v) used in the present experiments was 9:1, 17:3, 4:1, and 3:1, respectively. 1D BN microtubes were also synthesized by adding 1 mL of a stock solution of BN in THF (2.5 mM) into 5 mL of (v/v) ethanol/H_2_O mixtures. The volume ratio (v/v) was set to 4:1, 3:1, 7:3, and 13:7, respectively. The resultant colloidal samples were collected on the surface of a quartz substrate. Meanwhile, part of the colloid solution was separated by centrifugation at 1500 rpm and washed several times with ultrapure water, and finally dried under vacuum for further analysis.

### Synthesis of BA/BN helical microribbons

Similar to pure BA or BN assemblies, 1 mL of a stock solution of BA/BN in THF with m/m = 2:1 was rapidly injected into 5 mL of (v/v) an ethanol/H_2_O mixture at room temperature, and the mixture was allowed to stand for 10 min. Afterwards a brownish black suspension was formed. In the present system, BA/BN helices were formed at different volume ratio (v/v = 17:3, 4:1, 3:1, 7:3, and 13:7), and the concentrations of BA and BN in THF were set to 5 mM and 2.5 mM, respectively.

### Synthesis of single crystals of BN alloys **a**, **b**, **c** and **d**

Single crystal of pure BN was obtained by slow evaporation of a stock solution of BN in THF (5 mM). Similarly, single crystals of alloys **a** and **b** were achieved by slow evaporation of a stock solution of BA/BN in THF, while single crystals of alloys **c** and **d** were achieved in DMF. In the present alloy systems, the concentrations of BA and BN in THF and DMF were set to 5 mM and 2.5 mM, respectively.

### Synthesis of BA@(BA)_*x*_(BN)_1-*x*_ core-shell structures via a solute exchange process

Specifically, saturated supernatants of the as-prepared (BA)_0.72_(BN)_0.28_ alloy helices (1, 2, 3 mL) formed at m/m = 2:1 and v/v = 4:1 were mixed with the pure BA microtubes, respectively. In the present system, BA tubes act as a seed and a saturated supernatant of as-prepared (BA)_0.72_(BN)_0.28_ alloy helices as the shell layer solution. The mixtures were kept undisturbed for 2 h and then the resultant colloidal samples were collected on the surface of a quartz substrate.

### Sample characterization

The morphologies and sizes of the samples were examined using field-emission scanning electron microscopy (FESEM, FEI Quanta 200 F) at an acceleration voltage of 5 kV. Prior to analysis, the samples were coated with a thin gold layer using an Edwards Sputter Coater. TEM images were obtained using a Philips CM 200 electron microscope operated at 80 kV. One drop of the as-prepared colloidal dispersion was deposited on a carbon-coated copper grid, and dried under high vacuum. The X-ray diffraction (XRD) patterns were measured by a D/max 2400 × -ray diffractometer with Cu Kα radiation (λ = 1.54050 Å) operated in the 2θ range from 3 to 30^°^, by using the samples spin-coated on the surface of a quartz substrate. The PL spectra of the samples were measured on a HORIBA JOBTN YVON FLUOROMAX-4 spectrofluorimeter with a slit width of 1 nm. The samples were both deposited on the surface of a quartz substrate. The fluorescence microscopy images were obtained using a Leica DMRBE fluorescence microscopy with a spot-enhanced charged couple device (CCD, Diagnostic Instrument, Inc.). Especially, blue (430-460 nm) and green light (510-550 nm) was used as the excitation light sources, respectively. The samples were prepared by placing a drop of dispersion onto a cleaned quartz slide. Real-time videos were captured by in-vivo tracking the formation processes of helical microribbons using laser confocal scanning microscopy (Leica, TCS-SP5) equipped with a green laser (543 nm).

## Electronic supplementary material

Supplementary Information

Description of Additional Supplementary Files

Supplementary Movie 1

Supplementary Movie 2

Supplementary Movie 3

## Data Availability

All relevant data supporting the findings of this study are available from the corresponding author on request.
