# Supplementary material for: Complex assembly from planar and twisted π-conjugated molecules towards alloy helices and core-shell structures
Source: Nat Commun. 2018 Oct 19;9:4358. doi: 10.1038/s41467-018-06489-3 (PMC6195596; doi:10.1038/s41467-018-06489-3)
Supplement: Supplementary file 1 — Supplementary Information [file 41467_2018_6489_MOESM1_ESM.pdf]

Supplementary Information

Complex assembly from planar and twisted π-conjugated molecules towards alloy helices and core-shell structures

Lei et al.

Supplementary Methods

Synthesis of $(BA)_x(BN)_{1-x}$ alloy assemblies formed at different molar ratio (m/m)

Typically, 1 mL of stock solutions of BA/BN in THF with different molar ratio (m/m = 100:1, 20:1, 4:1, 4:3, and 1:1) were rapidly injected into 5 mL of (v/v) ethanol/H_2O mixtures. In our present systems, the concentration of BA in THF (C_{BA} = 5 mM) was held constant. To ensure the consistency of morphologies and sizes of the samples in the present system, the volume ratio of ethanol and H_2O (v/v) should be adjusted rationally. For instance, v/v was set to 9:1 at m/m = 100:1 and 20:1, whereas it was changed to 17:3 at m/m = 4:1, 3:1 at m/m = 4:3, and 7:3 at m/m = 1:1. The dependence of BA/BN co-assemblies on volume ratio (v/v) and molar ratio in THF solution (m/m) was summarized in Supplementary Table 1. All the mixtures were kept undisturbed for 10 min, afterwards five types of suspensions with different color changes from orange to brownish black were formed. Similarly, the resultant colloidal samples were collected on the surface of a quartz substrate. Meanwhile, parts of the colloid solutions were separated by centrifugation at 3000 rpm and washed several times with ultrapure water, and finally dried under vacuum for further analysis.

Co-assembly of BA and rubrene

Pure BA microtubes were obtained by mixing 1 mL of a stock solution of BA in THF (C_{BA} = 20 mM) with 5 mL of methanol. Similarly, irregular rubrene rods were also achieved by mixing 1 mL of a stock solution of rubrene in THF ($C_{rubrene}$ = 10 mM) with 5 mL of methanol. The co-assembly of BA and rubrene was performed by mixing 1 mL of a stock solution of BA/rubrene in THF with 5 mL of methanol. In the present co-assembly systems, the concentrations of BA and rubrene in THF were set to 20 mM and 10 mM, respectively.

Theoretical calculation

The theoretical calculation was performed using Gaussion09 D01 package. To get more information about the molecular orientation of BN in the BA crystal, an ONIOM (B3LYP/6-31G (d,p):UFF) approach was used to optimize the geometries built by doping BN into BA crystal (BN doped BA). The solid phase calculation of BN doped BA was achieved using the ONIOM model, with one central BA molecule as high layer calculated using QM method with DFT calculations at the B3LYP/6-31G(d,p) level and all the surrounding BN molecules as low layer calculated using MM method with the UFF

force field. The BN doped BA cluster is built as a $2\times4\times4$ BA supercell based on the unit cell of BA, with one central BA molecule replaced by a BN molecule. Since all the BA molecules are equivalent in the crystal, only one position needs to be replaced. In the ONIOM calculations, the active region is optimized and the environmental region is frozen. The active region includes the BN molecule and eight surrounding BA molecules. Similarly, the BA doped BN cluster was also built as a $2.5\times1.3\times2.5$ BN supercell based on the unit cell of BN, with one central BN molecule replaced by a BA molecule. The active region of the BA doped BN cluster includes the BA molecule and eight surrounding BN molecules.

Supplementary Figures:

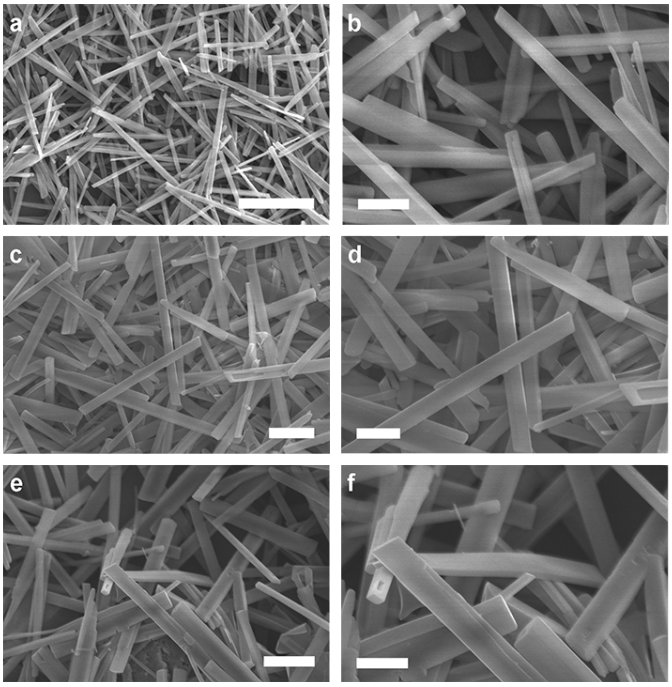

Supplementary Figure 1. SEM images of 1D BA assemblies formed by adding a stock solution of pure BA in THF (1 mL) into 5 mL of ethanol/H_2O mixtures at v/v = **a**, **b** 17:3, **c**, **d** 4:1, and **e**, **f** 3:1 at **a**, **c** and **e** low and **b**, **d** and **f** high magnification. Scale bars, 25 μm in **a**, 5 μm in **b**, **d**, **f**, and 10 μm in **c**, **e**.

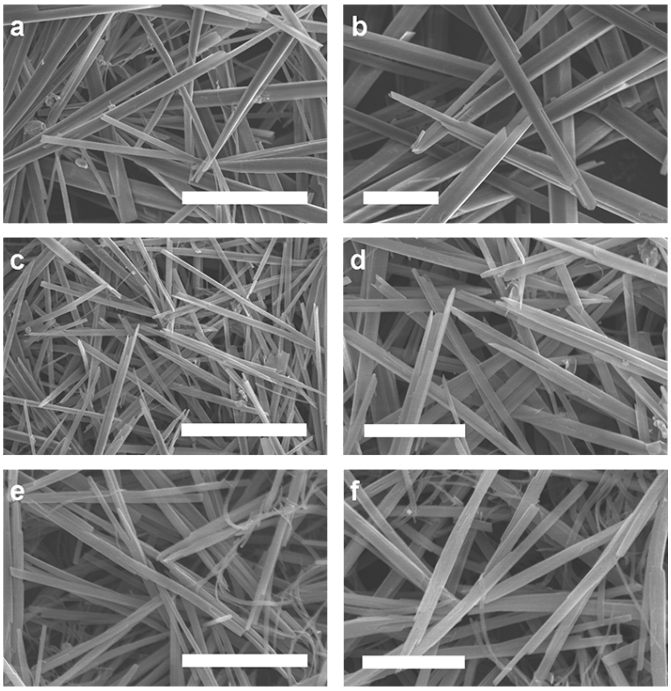

Supplementary Figure 2. SEM images of 1D BN assemblies formed by adding stock solutions of pure BN in THF (1 mL) into 5 mL of ethanol/H_2O mixtures at v/v = **a**, **b** 3:1, **c**, **d** 7:3, and **e**, **f** 13:7 at **a**, **c**, **e** low and **b**, **d**, **f** high magnification. Scale bars, 50 μm in **a**, **c**, 25 μm in **b**, 20 μm in **d**, **e**, and 10 μm in **f**.

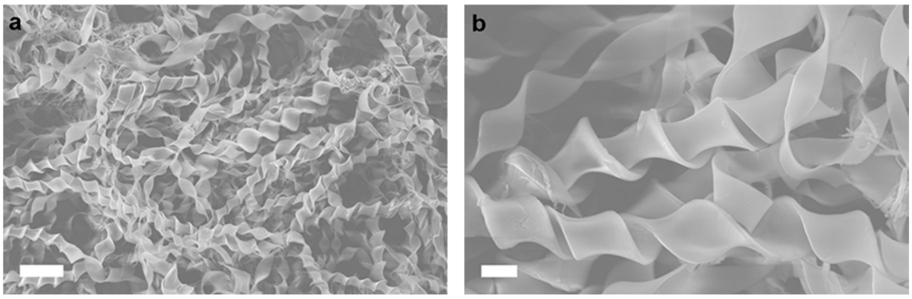

Supplementary Figure 3. SEM images of BA/BN helical ribbons deposited on a quartz substrate when exposed to daylight for two weeks at **a** low and **b** high magnification. Scale bars, 10 μm in **a** and 2 μm in **b**.

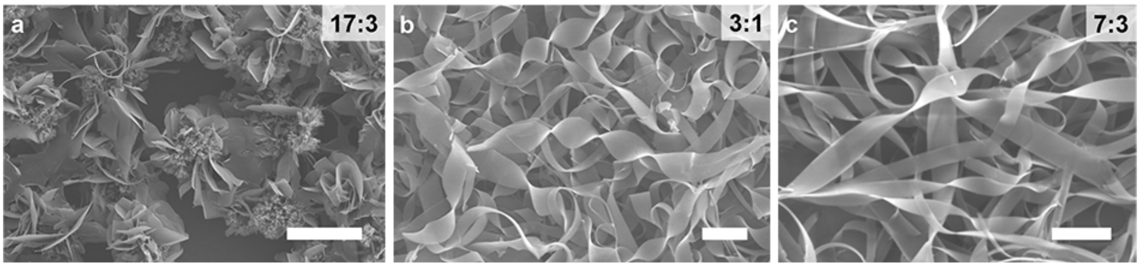

Supplementary Figure 4. SEM images of BA/BN helices formed by injecting stock solutions of BA/BN in THF (1 mL) with m/m = 2:1 into 5 mL of ethanol/H_2O mixtures at v/v = **a** 17:3, **b** 3:1, and **c** 7:3. Scale bars, 50 μm in **a** and 10 μm in **b** and **c**.

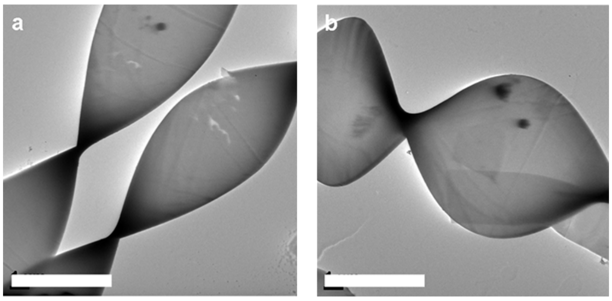

Supplementary Figure 5. a, **b** TEM images of typical twisted ribbons formed by injecting a stock solution of BA/BN in THF (1 mL) with m/m = 2:1 into 5 mL of an 3:1 ethanol/H_2O mixture. Scale bars, 5 μm.

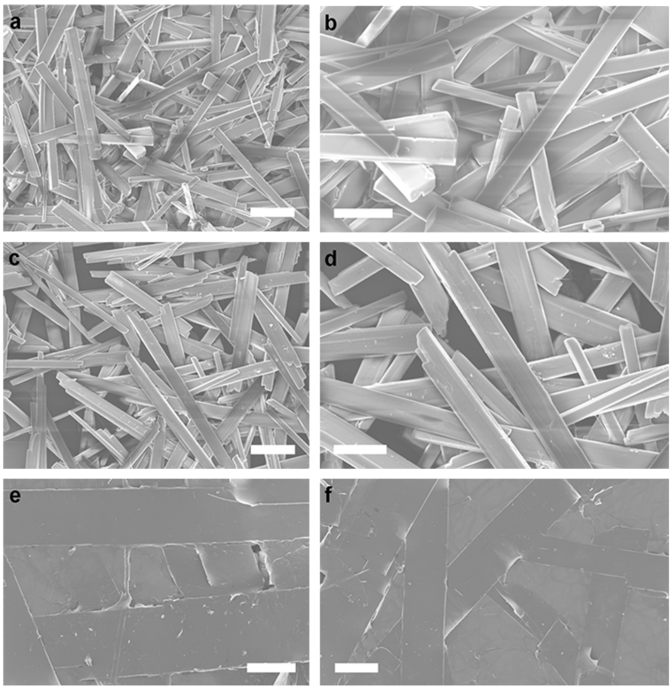

Supplementary Figure 6. SEM images of BA/BN co-assemblies obtained at m/m = **a**, **b** 100:1, **c**, **d** 20:1, and **e**, **f** 4:1 at **a**, **c**, **e** low and **b**, **d**, **f** high magnification. Scale bars, 20 μm in **a**, **c**, **e**, **f** and 10 μm in **b**, **d**.

Supplementary Table 1. Dependence of co-assembly morphologies of BA and BN and related optical properties on volume ratio (v/v) and molar ratio in THF solution (m/m)

Molar ratio in THF solution (m/m)	Volume ratio (v/v)	Morphologies	Emission colors	Exact molar ratio in co-assemblies (m/m)
100:0	9:1	tubes	yellow-green	1:0
100:1	9:1	tubes	orange-red	
20:1	9:1	tubes	red	0.94:0.06
4:1	17:3	straight ribbons	NIR	0.8:0.2
	17:3	flower-like helices	NIR	
	4:1	helical ribbons	NIR	0.72:0.28
2:1	3:1	twisted ribbons	NIR	
	7:3	partial helices	NIR	
	13:7	straight ribbons	NIR	
4:3	3:1	helical ribbons	NIR	
1:1	7:3	helical ribbons	NIR	
0:100	4:1	tubes	NIR	0:1

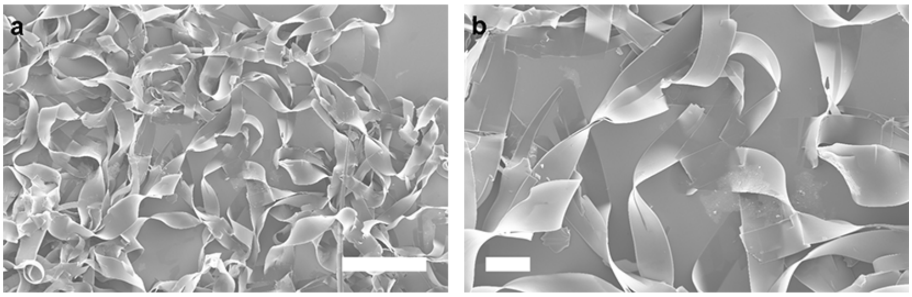

Supplementary Figure 7. SEM images of BA/BN twisted helices formed by injecting a stock solution of BA/BN in DMF (1 mL) with m/m = 2:1 into 5 mL of an 17:3 ethanol/H_2O mixture at **a** low and **b** high magnification. Scale bars, 100 μm in **a** and 20 μm in **b**.

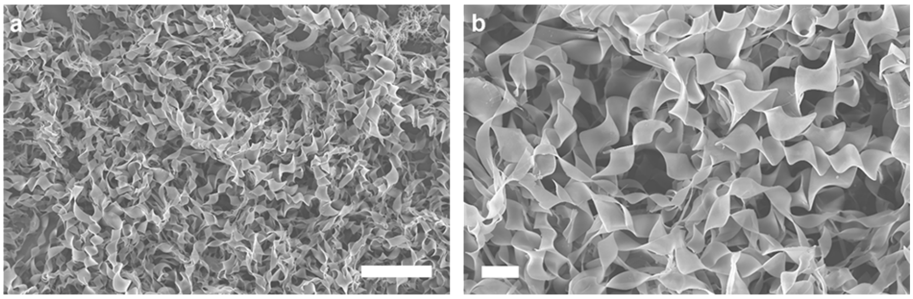

Supplementary Figure 8. SEM images of BA/BN helical ribbons formed by injecting a stock solution of BA/BN in acetone (1 mL) with m/m = 2:1 into 5 mL of an 4:1 ethanol/H_2O mixture at **a** low and **b** high magnification. Scale bars, 50 μm in **a** and 10 μm in **b**.

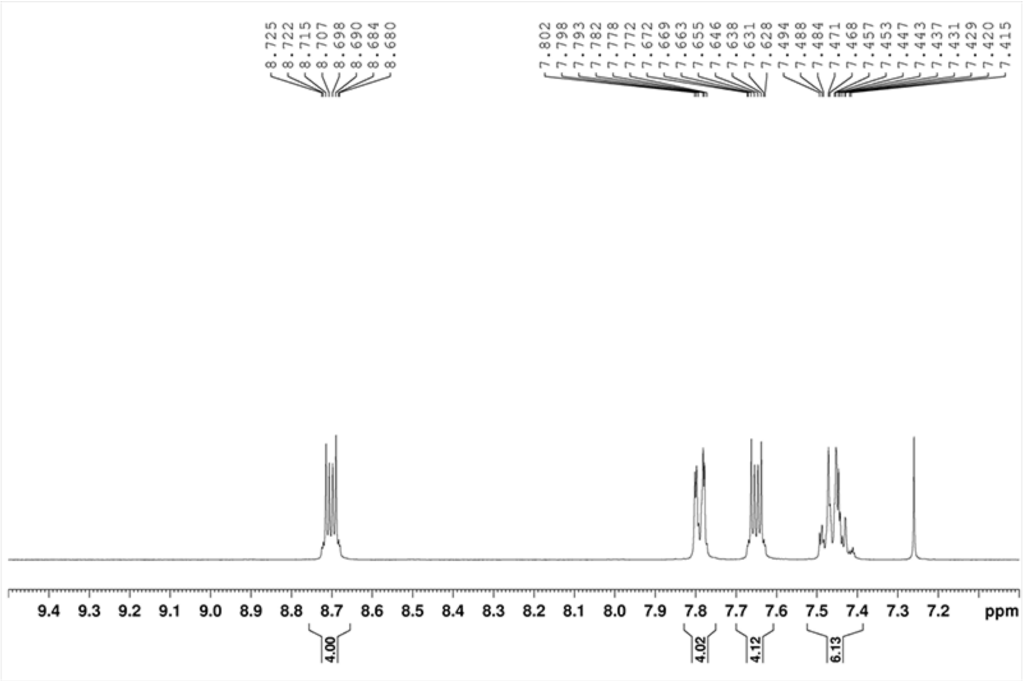

Supplementary Figure 9. ^{1}H NMR spectrum of BA microtubes.

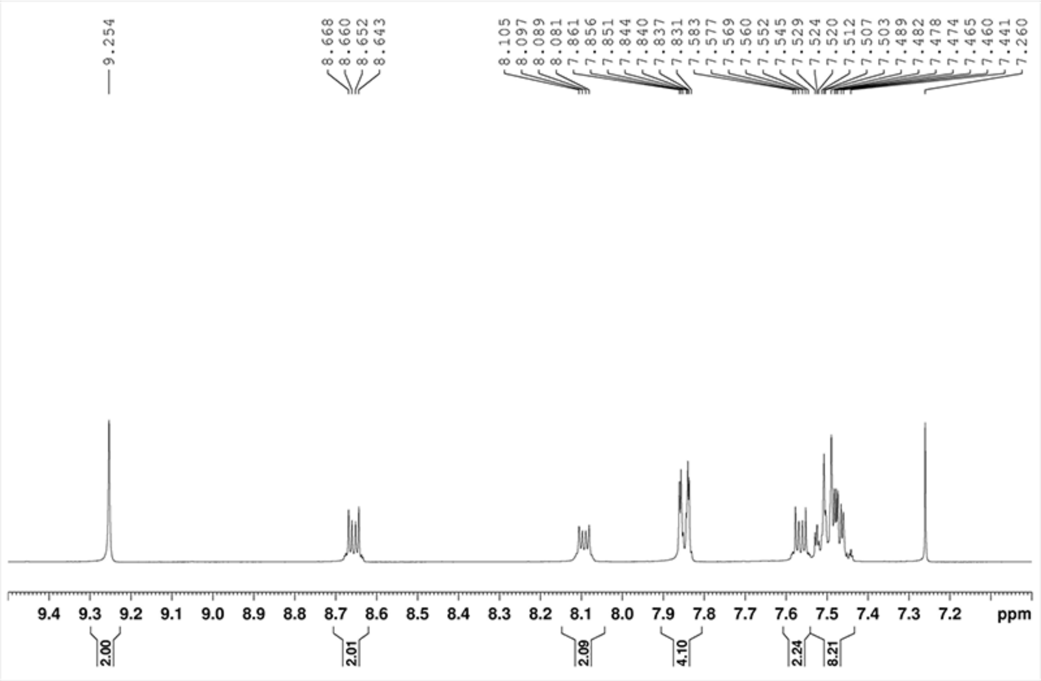

Supplementary Figure 10. ^{1}H NMR spectrum of BN microtubes.

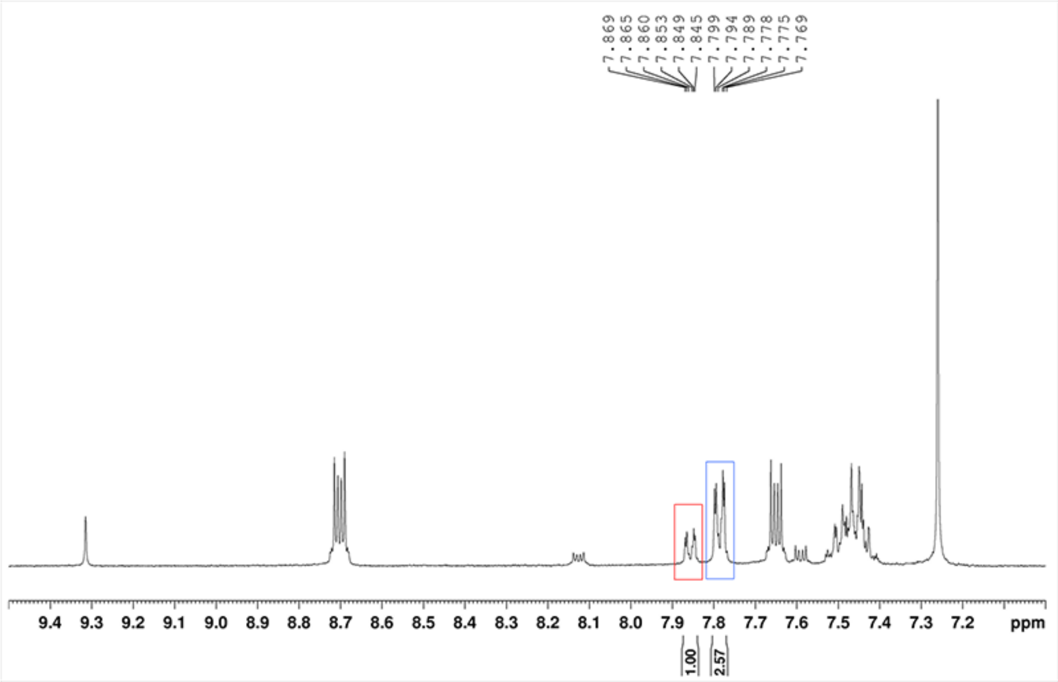

Supplementary Figure 11. ^{1}H NMR spectrum of BA/BN helical ribbons obtained by injecting a stock solution of 2:1 BA/BN in THF (1 mL) into 5 mL of 4:1 ethanol/H_2O mixture. The blue solid box represents the ^{1}H NMR spectra integral of BA component, whereas the red solid box exhibits the ^{1}H NMR spectra integral of BN component.

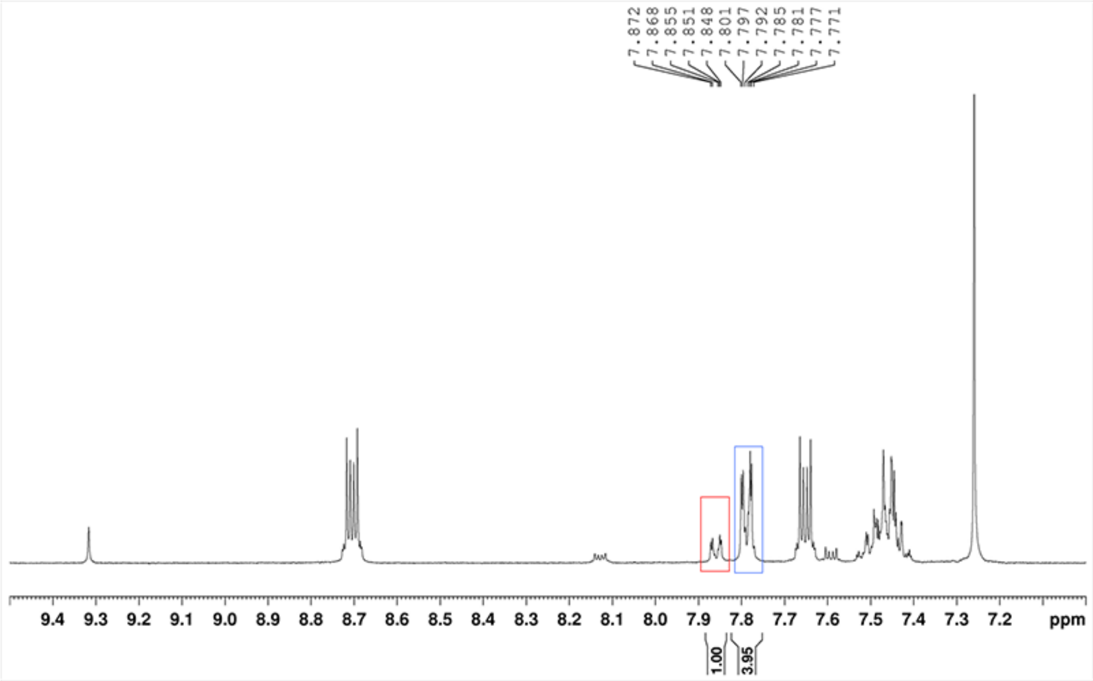

Supplementary Figure 12. ^{1}H NMR spectrum of BA/BN co-assemblies obtained at m/m = 4:1.

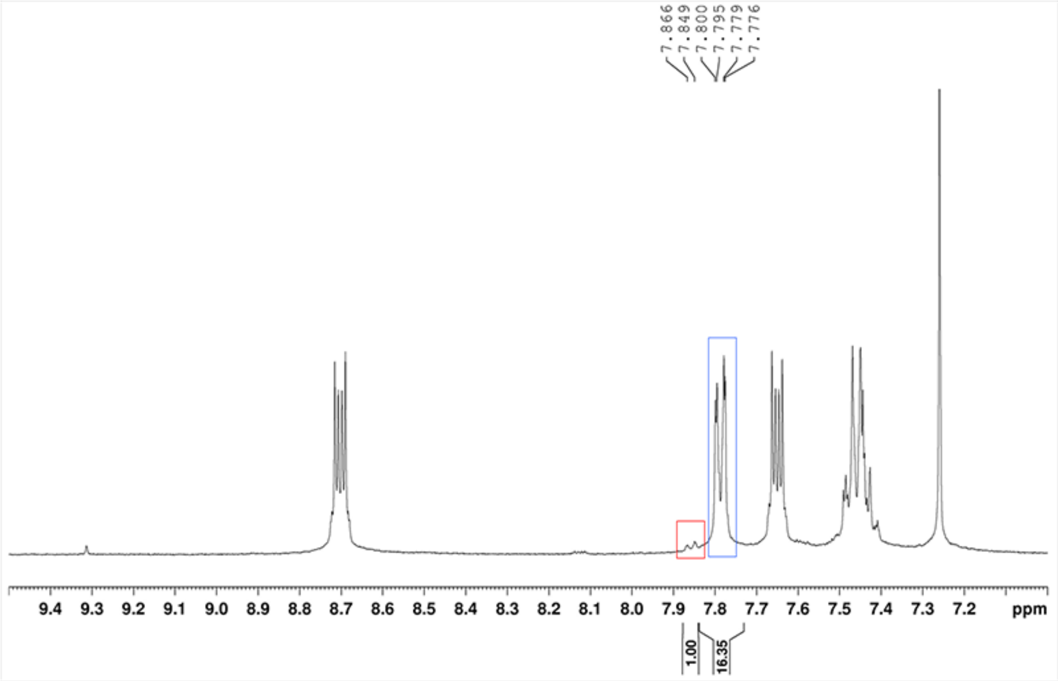

Supplementary Figure 13. ^{1}H NMR spectrum of BA/BN co-assemblies obtained at m/m = 20:1.

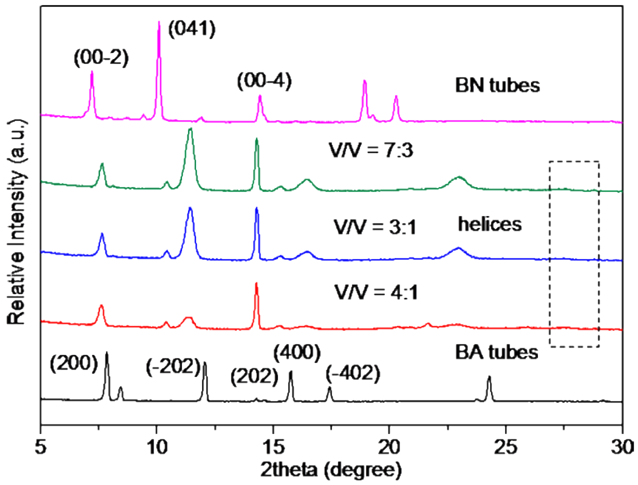

Supplementary Figure 14. Powder XRD patterns of BA tubes, BN tubes, and BA/BN helical ribbons formed by injecting stock solutions of BA and BN in THF (1 mL) with m/m = 2:1 into 5 mL of ethanol/H_2O mixtures at v/v = 4:1, 3:1, and 7:3.

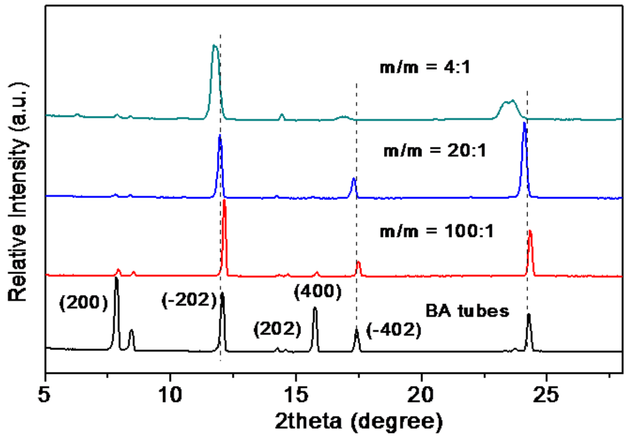

Supplementary Figure 15. Powder XRD patterns of BA microtubes and BA/BN co-assemblies obtained at m/m = 100:1, 20:1, and 4:1.

Supplementary Table 2. Summary of crystallographic data of BA, BN, and $(BA)_x(BN)_{1-x}$ alloys formed at m/m = 2:1 (Alloys **a** and **b** were grown from THF, whereas alloys **c** and **d** were obtained from DMF).

	BA	BN	Alloy **a**	Alloy **b**	Alloy **c**	Alloy **d**
Space Group	C 2/c	C 2/c	C 2/c	C 2/c	P-1	C 2/c
Cell Lengths (Å)	a = 22.866<br>b = 5.3567<br>c = 16.93	a = 10.126<br>b = 37.223<br>c = 24.495	a = 22.841<br>b = 5.3697<br>c = 16.953	a = 22.617<br>b = 5.358<br>c = 16.809	a = 5.537<br>b = 11.954<br>c = 17.501	a = 23.063<br>b = 5.3447<br>c = 17.062
Cell Angles	α = 90°<br>β = 99.72°<br>γ = 90°	α = 90°<br>β = 92.93°<br>γ = 90°	α = 90°<br>β = 100°<br>γ = 90°	α = 90°<br>β = 99.14°<br>γ = 90	α = 98.36°<br>β = 91.59°<br>γ = 102.44°	α = 90°<br>β = 100.6°<br>γ = 90°
Cell Volume (Å^3)	2043.92	9220.59	2047.66	2011.08	1117.07	2067.25

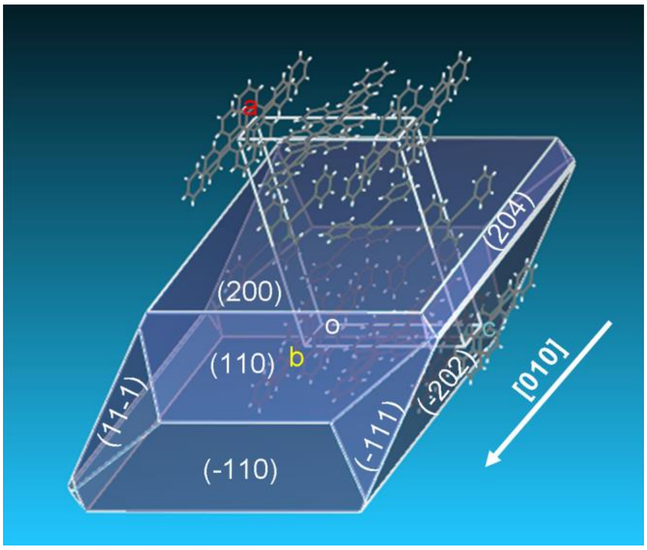

Supplementary Figure 16. The predicted growth morphology and direction of BA crystal simulated by Material Studio based on the attachment energies.

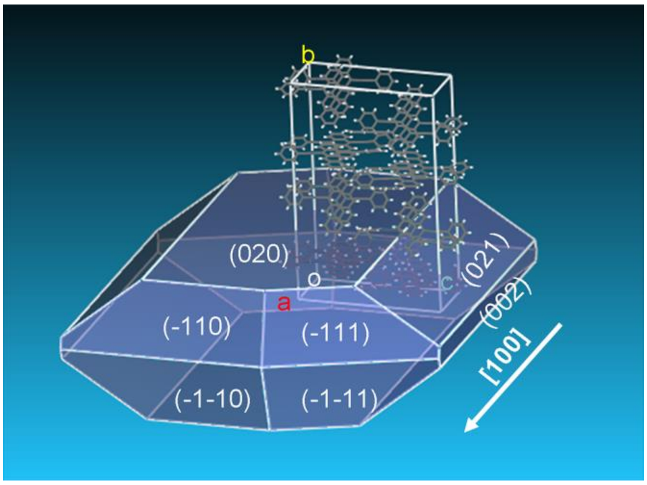

Supplementary Figure 17. The predicted growth morphology and direction of BN crystal simulated by Material Studio based on the attachment energies.

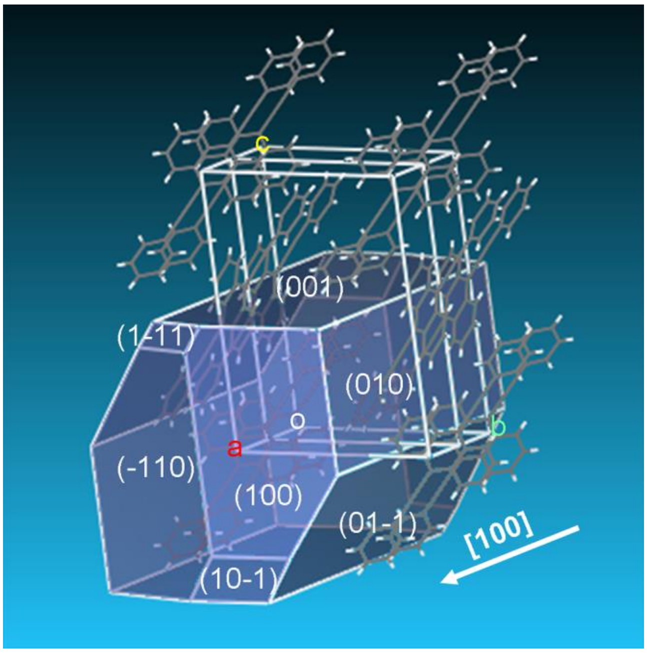

Supplementary Figure 18. The predicted growth morphology and direction of alloy **c** simulated by Material Studio based on the attachment energies.

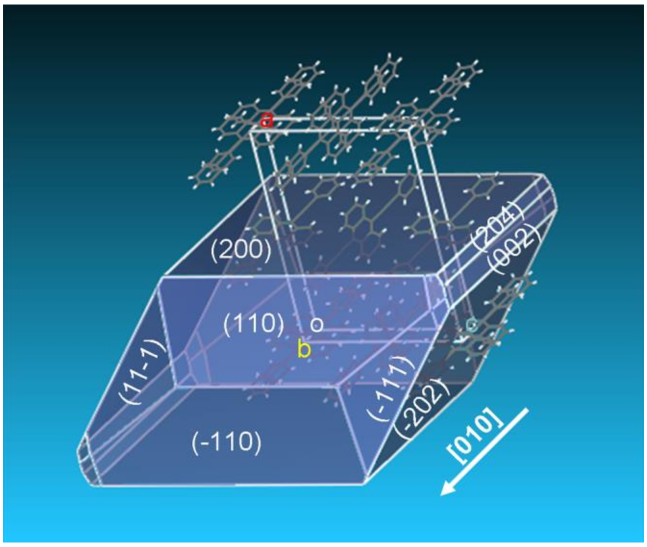

Supplementary Figure 19. The predicted growth morphology and direction of alloy **d** simulated by Material Studio based on the attachment energies.

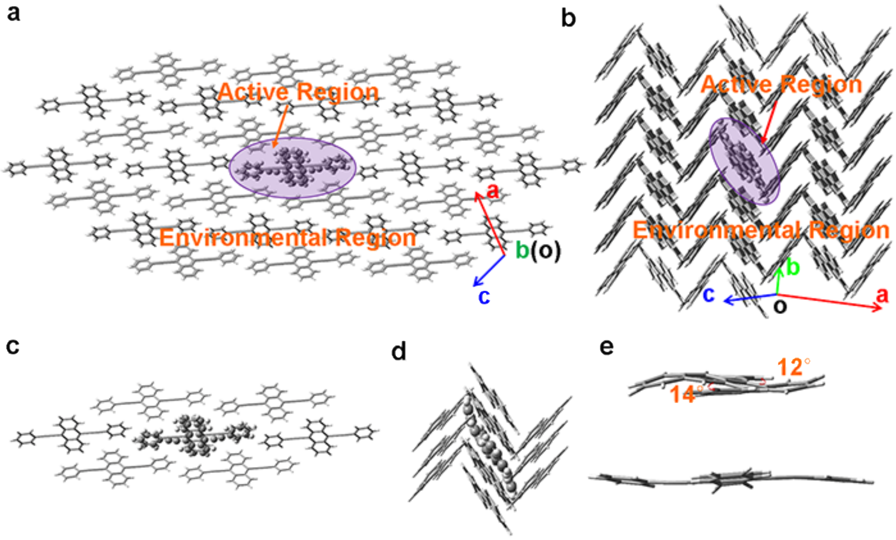

Supplementary Figure 20. a, **b** ONIOM model of BN doped BA: single BN molecule is treated quantum mechanically as the center of active region; its eight nearest molecules are optimized at the MM level; and the remaining molecules are fixed at the MM level as environment. **c**, **d** The local structure of active region. **e** The torsion angles between the naphthacene and two phenylethynyl groups of BN molecule.

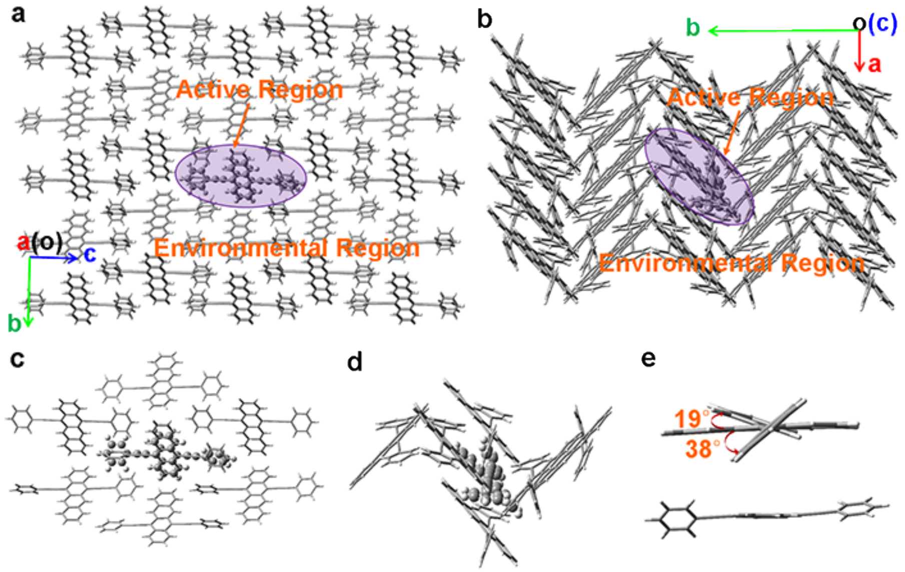

Supplementary Figure 21. **a**, **b** ONIOM model of BA doped BN: single BA molecule is treated quantum mechanically as the center of active region; its eight nearest molecules are optimized at the MM level; and the remaining molecules are fixed at the MM level as environment. **c**, **d** The local structure of active region. **e** The torsion angles between the anthracene and two phenylethynyl groups of BA molecule.

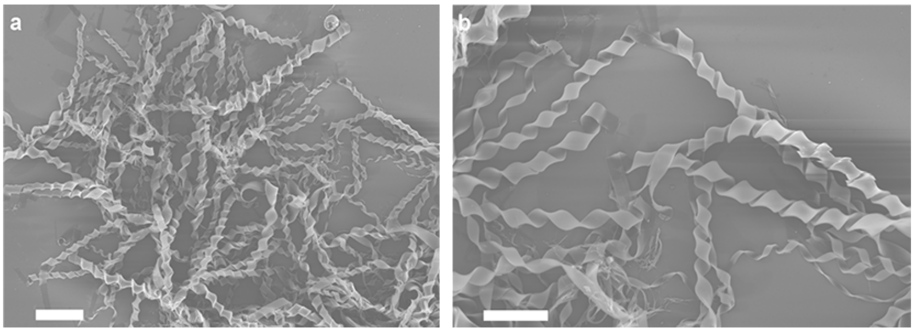

Supplementary Figure 22. SEM images of $(BA)_x(BN)_{1-x}$ helical ribbons formed by injecting a stock solution of BA/BN in THF (1 mL) with m/m = 4:3 into 5 mL of an 3:1 ethanol/H_2O mixture at **a** low and **b** high magnification. Scale bars, 20 μm in **a** and 10 μm in **b**.

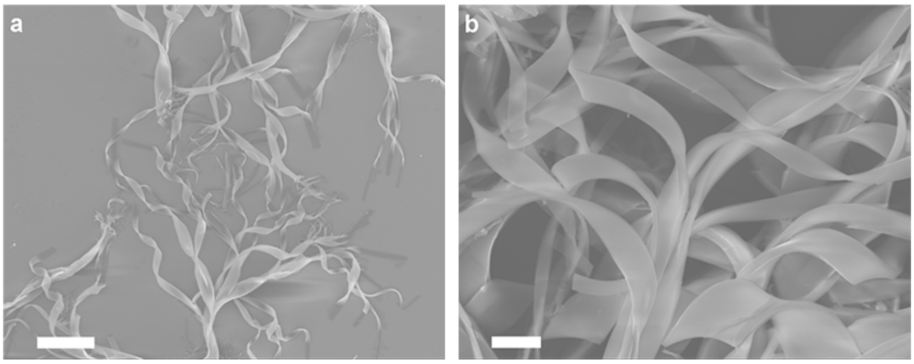

Supplementary Figure 23. SEM images of $(BA)_x(BN)_{1-x}$ helical ribbons formed by injecting a stock solution of BA/BN in THF (1 mL) with m/m = 1:1 into 5 mL of an 7:3 ethanol/H_2O mixture at **a** low and **b** high magnification. Scale bars, 10 μm in **a** and 2 μm in **b**.

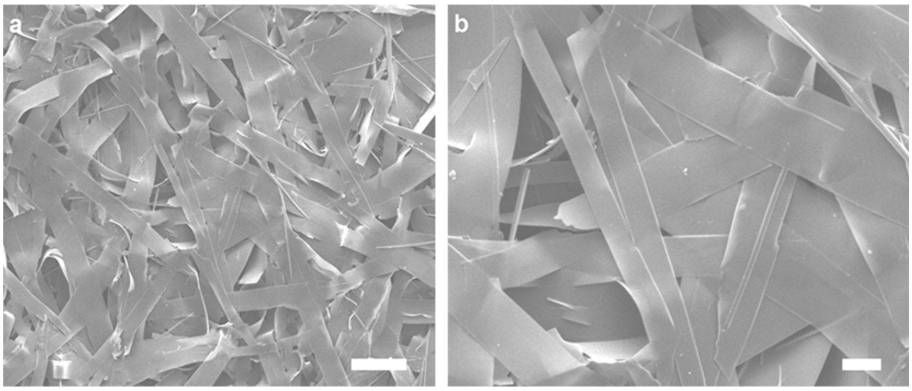

Supplementary Figure 24. SEM images of $(BA)_x(BN)_{1-x}$ alloy assemblies formed by injecting a stock solution of BA/BN in THF (1 mL) with m/m = 2:1 into 5 mL of an 13:7 ethanol/H_2O mixture at **a** low and **b** high magnification. Scale bars, 50 μm in **a** and 10 μm in **b**.

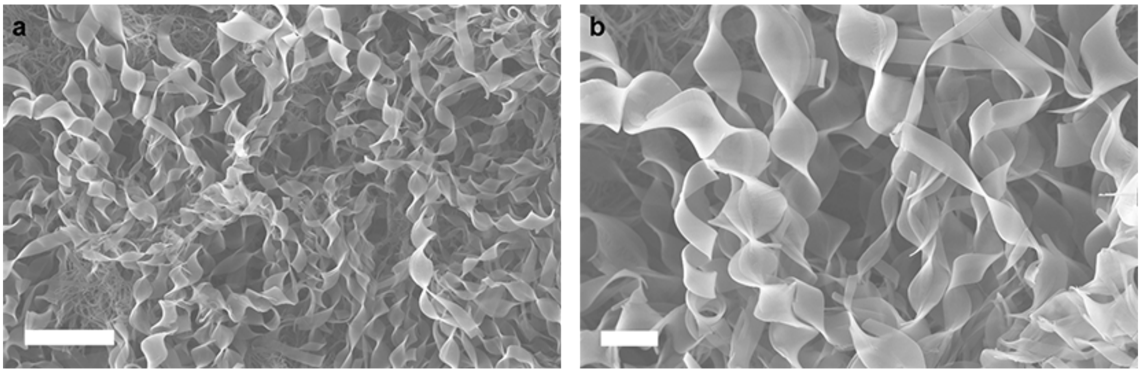

Supplementary Figure 25. SEM images of $(BA)_x(BN)_{1-x}$ helical ribbons formed by mixing lower concentrations of BA and BN in THF with a 2:1 molar ratio (C_{BA} = 2.5 mM, C_{BN} = 1.25 mM) with 5 mL of an 13:7 ethanol/H_2O mixture at **a** low and **b** high magnification. Scale bars, 20 μm in **a** and 5 μm in **b**.

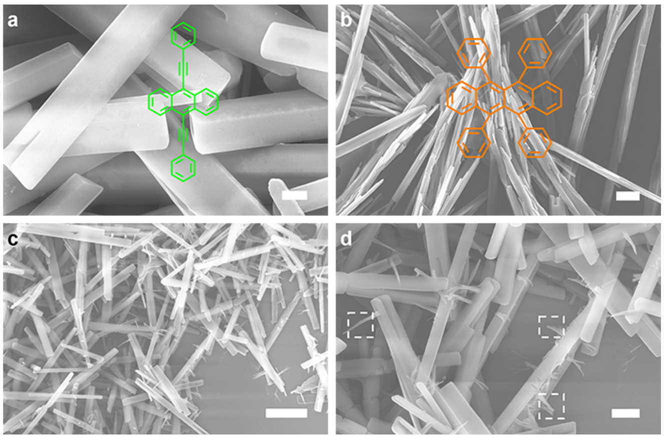

Supplementary Figure 26. SEM images of **a** BA tubes, **b** irregular rubrene rods, and **c**, **d** BA/rubrene mixtures formed by injecting a stock solution of BA/rubrene in THF (1 mL) with m/m = 2:1 into 5 mL of methanol. The dashed boxes represent short rubrene rods. Scale bars, 2 μm in **a**, 20 μm in **b**, **c**, and 5 μm in **d**.

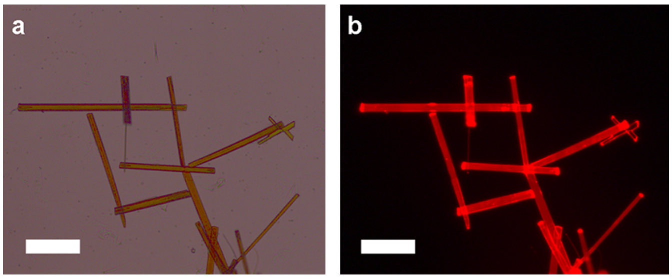

Supplementary Figure 27. **a** Bright-field optical and **b** fluorescence microscopy images of $(BA)_x(BN)_{1-x}$ alloy assemblies obtained at m/m = 100:1 when excited by **b** blue light, respectively. Scale bars, 50 μm.

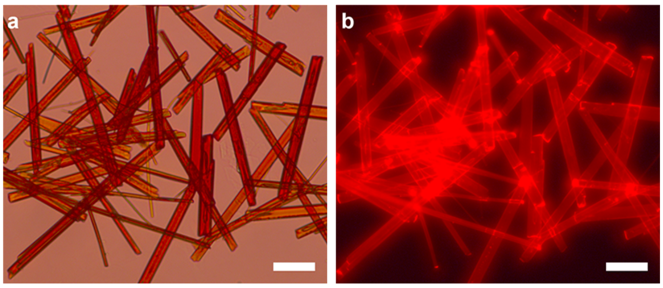

Supplementary Figure 28. **a** Bright-field optical and **b** fluorescence microscopy images of $(BA)_{0.94}(BN)_{0.06}$ alloy assemblies obtained at m/m = 20:1 when excited by **b** blue light, respectively. Scale bars, 25 μm.

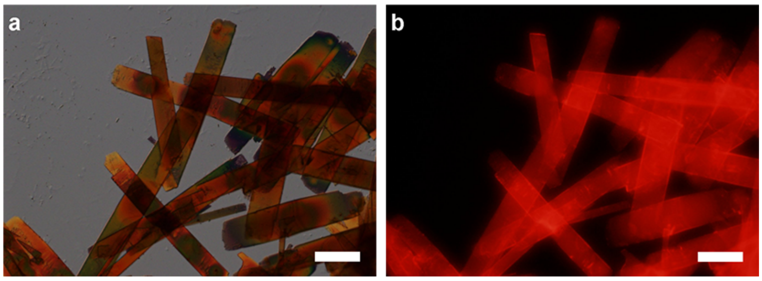

Supplementary Figure 29. a Bright-field optical and **b** fluorescence microscopy images of $(BA)_{0.8}(BN)_{0.2}$ alloy assemblies obtained at m/m = 4:1 when excited by **b** blue light, respectively. Scale bars, 50 µm.

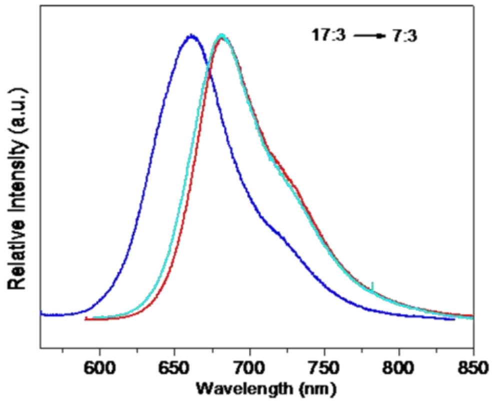

Supplementary Figure 30. PL spectra of BA/BN helices formed by injecting stock solutions of BA/BN in THF (1 mL) with m/m = 2:1 into 5 mL of ethanol/H_2O mixtures at v/v = 17:3, 3:1, and 7:3, respectively.

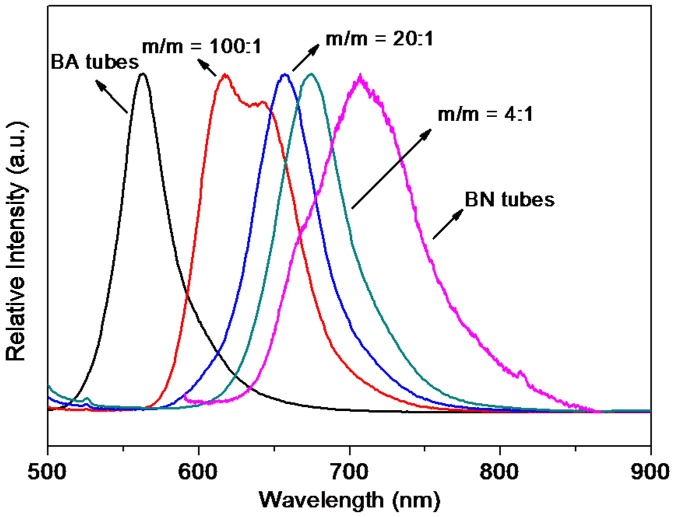

Supplementary Figure 31. PL spectra of BA tubes, BN tubes, and $(BA)_x(BN)_{1-x}$ alloy assemblies obtained at m/m = 100:1, 20:1, and 4:1. The excitation wavelength is 480 nm.

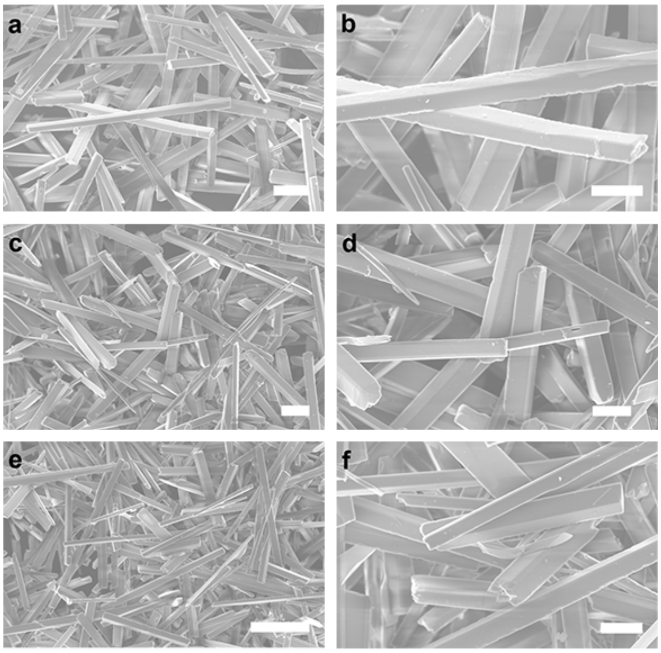

Supplementary Figure 32. SEM images of BA@$(BA)_x(BN)_{1-x}$ core-shell microstructures at **a**, **c**, **e** low and **b**, **d**, **f** high magnification obtained by mixing BA microtubes with **a**, **b** 1, **c**, **d** 2, and **e**, **f** 3 mL of saturated supernatants of $(BA)_{0.72}(BN)_{0.28}$ helices, respectively. Scale bars, 20 μm in **a**, **c**, 10 μm in **b**, **d**, **f**, and 50 μm in **e**.

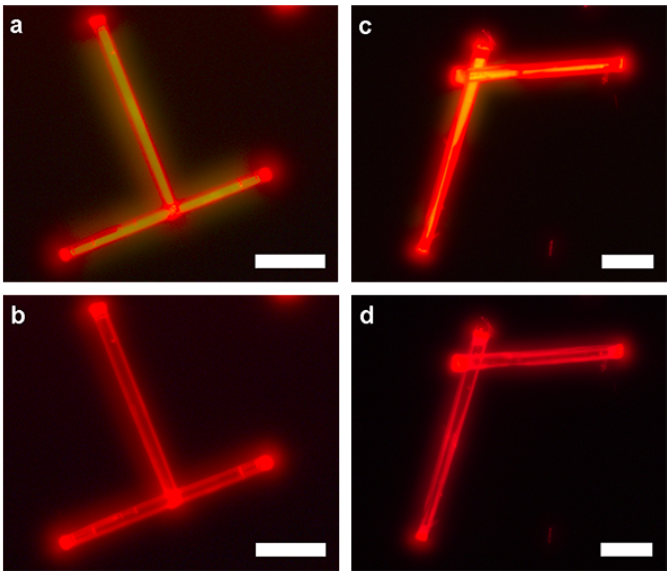

Supplementary Figure 33. Upon excitation with **a**, **c** blue and **b**, **d** green light, fluorescence microscopy images of BA@$(BA)_x(BN)_{1-x}$ core-shell microstructures obtained by mixing pure BA microtubes with **a**, **b** 1, and **c**, **d** 3 mL of saturated supernatants of $(BA)_{0.72}(BN)_{0.28}$ helices, respectively. Scale bars, 25 μm.

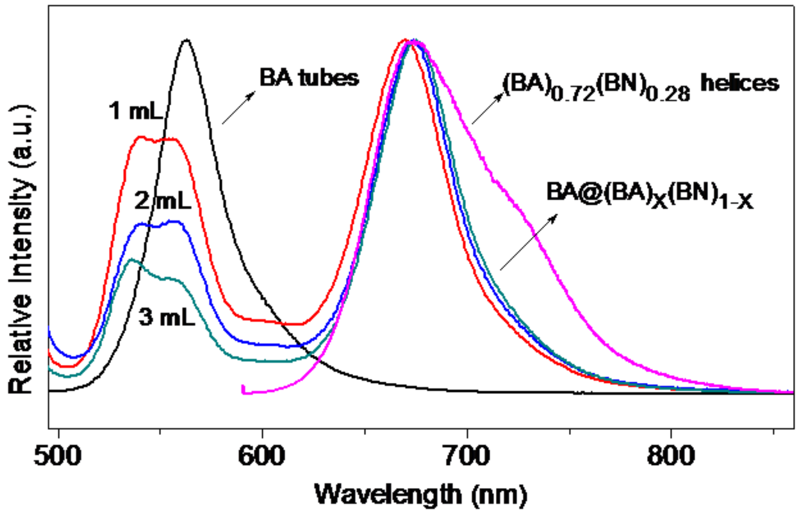

Supplementary Figure 34. PL spectra of BA tubes, $(BA)_{0.72}(BN)_{0.28}$ helices, and $BA@(BA)_x(BN)_{1-x}$ core-shell microstructures obtained by mixing BA tubes with 1, 2, and 3 mL of saturated supernatants of $(BA)_{0.72}(BN)_{0.28}$ helices, respectively. The excitation wavelength is 480 nm.

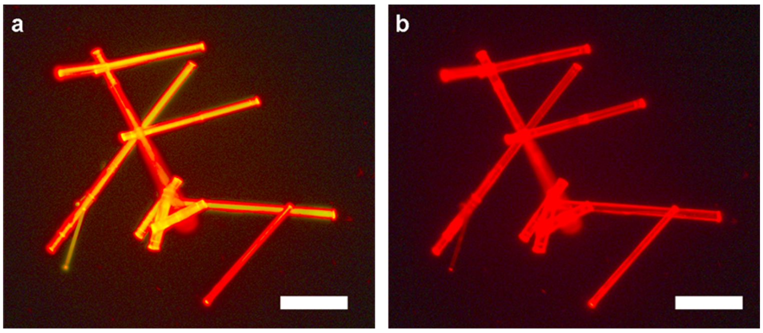

Supplementary Figure 35. Fluorescence microscopy images of $BA@(BA)_x(BN)_{1-x}$ core-shell microstructures obtained by mixing pure BA microtubes with 3 mL of a saturated supernatant of $(BA)_{0.94}(BN)_{0.06}$ alloy assemblies obtained at m/m = 20:1 when excited by **a** blue and **b** green light, respectively. Scale bars, 50 μm.

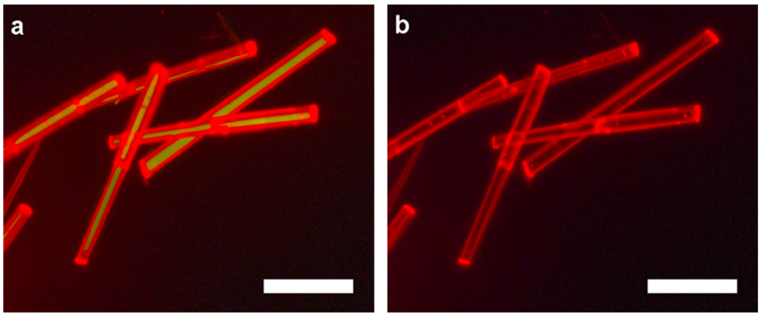

Supplementary Figure 36. Fluorescence microscopy images of BA@$(BA)_x(BN)_{1-x}$ core-shell microstructures obtained by mixing pure BA microtubes with 3 mL of a saturated supernatant of $(BA)_{0.8}(BN)_{0.2}$ alloy assemblies obtained at m/m = 4:1 when excited by **a** blue and **b** green light, respectively. Scale bars, 50 μm.

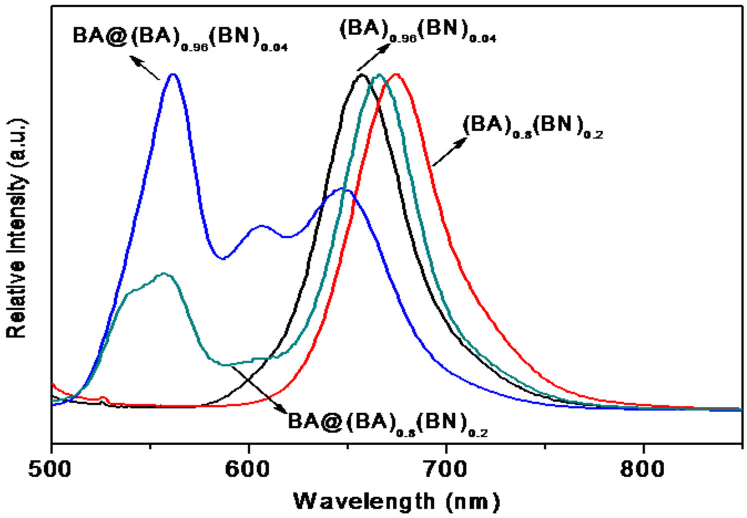

Supplementary Figure 37. PL spectra of $(BA)_{0.96}(BN)_{0.04}$, $(BA)_{0.8}(BN)_{0.2}$, and BA@$(BA)_x(BN)_{1-x}$ core-shell microstructures obtained by mixing pure BA microtubes with 3 mL of saturated supernatants of $(BA)_x(BN)_{1-x}$ alloy assemblies obtained at m/m = 20:1 ($(BA)_{0.96}(BN)_{0.04}$) and 4:1($(BA)_{0.8}(BN)_{0.2}$), respectively. The excitation wavelength is 480 nm.

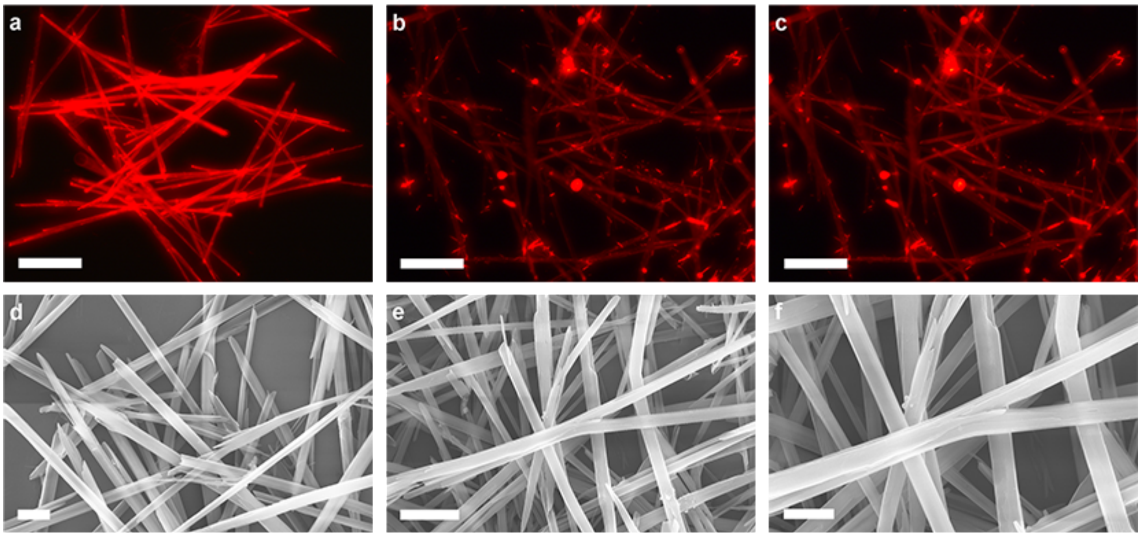

Supplementary Figure 38. Upon excitation with **a**, **b** blue and **c** green light, fluorescence microscopy images of **a** pure BN tubes and **b**, **c** mixtures of BN tubes formed by mixing pure BN microtubes with 3 mL of a saturated supernatant of $(BA)_{0.72}(BN)_{0.28}$ helices. Scale bars, 50 μm. SEM images of **d** pure BN tubes and **e**, **f** mixtures of BN tubes at **e** low and **f** high magnification. Scale bars, 10 μm in **d**, **f**, and 20 μm in **e**.
