# Supplementary material for: Complex assembly from planar and twisted π-conjugated molecules towards alloy helices and core-shell structures
Source: Nat Commun. 2018 Oct 19;9:4358. doi: 10.1038/s41467-018-06489-3 (PMC6195596; doi:10.1038/s41467-018-06489-3)
Supplement: Supplementary file 2 — Description of Additional Supplementary Files [file 41467_2018_6489_MOESM2_ESM.pdf]

Description of Additional Supplementary Files

Supplementary Movie 1

Description: In vivo imaging of the formation of helical ribbons.

Supplementary Movie 2

Description: In vivo imaging of the formation of helical ribbons.

Supplementary Movie 3

Description: In vivo imaging of the formation of helical ribbons.
